# Prognostic Impact of Recreational Drug Use on 1-Year Outcomes in NSTEMI and STEMI Patients

**DOI:** 10.1016/j.jacadv.2025.102574

**Published:** 2026-01-23

**Authors:** Michael Aboujaoude, Nefissa Hamache, Jean Guillaume Dillinger, Antonin Trimaille, Claire Bouleti, Clément Delmas, Guillaume Schurtz, Sonia Houssany-Pissot, Reza Rossanaly Vasram, Guillaume Bonnet, Damien Millischer, Christophe Thuaire, François Roubille, Nathalie Noirclerc, Stéphane Andrieu, Charles Fauvel, Raphael Mirailles, Julien Hudelo, Trecy Gonçalves, Jeremy Florence, Alexandre Unger, Manveer Singh, Emmanuel Gall, Alexandre Lafont, Solenn Toupin, Lynette Menezes, Eric Vicaut, Patrick Henry, Théo Pezel, ADDICT-ICCU Investigators

**Affiliations:** a Université Paris Cité, Service de Cardiologie, Hôpital Lariboisière, Assistance Publique-Hôpitaux de Paris, Paris, France; b Université Paris Cité, Inserm MASCOT-UMRS 942, University Hospital of Lariboisiere, Paris, France; c USF Health Morsani College of Medicine, University of South Florida, Tampa, Florida, USA; d Department of Cardiology, Centre Hospitalier Régional Universitaire (CHRU)-Nancy, Université de Lorraine, Nancy, France; e Imagerie Adaptative Diagnostique et Interventionnelle (IADI), INSERM, U1254, Université de Lorraine, Nancy, France; f Département de Cardiologie, Centre Hospitalier Universitaire (CHU de Strasbourg), Strasbourg, France; g Department of Cardiology, University Hospital of Poitiers, Poitiers, France; h Cardiology Department, Rangueil University Hospital, Toulouse, France; i Department of Cardiology, University Hospital of Lille, Lille, France; j Department of Cardiology, Military Hospital Percy, Clamart, France; k Department of Cardiology, Felix-Guyon University Hospital, Saint-Denis-de-La-Reunion, France; l Cardiology Intensive Care Unit and Interventional Cardiology, Hôpital Cardiologique du Haut-Lévêque, Pessac, France; m Bordeaux Cardiothoracic Research Center, U1045, Bordeaux University, Bordeaux, France; n Service de Cardiologie, Hôpital Montfermeil, Montfermeil, France; o Service de Cardiologie, Centre Hospitalier de Chartres, Le Coudray, France; p Service de Cardiologie, CHU Montpellier, Montpellier, France; q Service de Cardiologie, Centre Hospitalier Annecy Genevois, Epagny Metz-Tessy, France; r Service de Cardiologie, Hôpital Henri Duffaut, Avignon, France; s Université Rouen Normandie, Inserm U1096, CHU Rouen, Department of Cardiology, Rouen, France; t Département de Cardiologie, Centre Hospitalier Universitaire (CHU Amiens), Amiens, France; u Cardiology Department, CHU Clermont-Ferrand, Clermont-Ferrand, France; v Université Libre de Bruxelles (ULB), Départment de Cardiologie, CUB Hôpital Erasme, Bruxelles, Belgium; w Université Paris Cité, Service de Cardiologie, Hôpital Cochin, Assistance Publique-Hôpitaux de Paris, Paris, France; x Unité de Recherche Clinique, AP-HP Nord, Paris, France

**Keywords:** acute coronary syndrome, major adverse cardiovascular events, recreational drug use, ST-segment elevation myocardial infarction

## Abstract

**Background:**

Recreational drug use is increasingly associated with adverse outcomes in acute coronary syndrome (ACS) patients, but differences in long-term outcomes between ST-segment elevation myocardial infarction (STEMI) and non-ST-segment elevation myocardial infarction are not well defined.

**Objective:**

The authors evaluated the association between recreational drug use and major adverse cardiovascular events (MACE) 1 year after intensive cardiac care unit (ICCU) admission in ACS patients.

**Methods:**

The Addiction in Intensive Cardiac Care Units study systematically screened all patients admitted to ICCUs across 39 French centers (April 7-22, 2021) via prospective urinary testing. The primary outcome was MACE, defined as cardiovascular death, nonfatal myocardial infarction, or stroke. One-year follow-up was collected through clinical visits or direct contact between patients and cardiologists, concluding in June 2022. Outcomes were adjudicated by an independent cardiology committee. The prognostic impact of recreational drug use on MACE was assessed using multivariable Cox proportional hazards models, validated by propensity matching.

**Results:**

Of 712 ACS patients, 13.5% had recreational drug detection. At 1 year, MACE occurred in 7.0%, with higher rates among drug-positive vs drug-negative patients (12.5% vs 6.2%). Recreational drug use was associated with increased MACE (HR: 2.70; 95% CI: 1.30-5.57; *P* = 0.013). This association was significant in STEMI (HR: 4.11; 95% CI: 1.60-10.5; *P* = 0.005) but not in non-ST-elevation myocardial infarction patients. Propensity matching confirmed this in STEMI patients (HR: 3.39; 95% CI: 1.19-9.62; *P* = 0.022).

**Conclusions:**

Recreational drug use was associated with increased 1-year MACE risk in ACS patients, particularly STEMI, supporting routine drug screening.

Acute coronary syndrome (ACS), encompassing non-ST-segment elevation myocardial infarction (NSTEMI) and ST-segment elevation myocardial infarction (STEMI), remains one of the leading causes of mortality worldwide despite advancements in diagnoses and treatments.^1^^,^^2^ ACS refers to a spectrum of clinical conditions resulting from acute myocardial ischemia caused by a sudden reduction in coronary blood flow. While traditional risk factors such as hypertension, diabetes, and dyslipidemia are well-established, new evidence points to recreational drug use as a prognostic factor in ACS patients, potentially influencing outcomes differently in NSTEMI and STEMI subgroups. Several studies have demonstrated that recreational drugs, such as cocaine and cannabis, leads to worse cardiovascular outcomes.^3^, ^4^, ^5^, ^6^ One study found that 20% of young ACS patients who used recreational drugs exhibited larger myocardial infarctions and reduced left ventricular function compared to nonusers.^7^ Similarly, a Mediterranean cohort found that 6.8% of ACS patients under 50 years of age had a positive urine test for cocaine, underscoring the relevance of drug use in younger ACS populations.^8^

Our research team has explored this topic through the ADDICT-ICCU (Addiction in Intensive Cardiac Care Units) study, a multicenter cohort of consecutive intensive cardiac care unit (ICCU) patients across France. Drug use was independently associated with an increased risk of in-hospital adverse events in all ICCU patients, with an adjusted odds ratio of 8.84 compared to nonusers (95% confidence interval [CI] 4.68-16.7, *P* < 0.001).^9^ Furthermore, in ICCU patients with STEMI, drug detection was independently associated with a significantly higher risk of major adverse cardiac events (MACE), including ventricular arrhythmias.^10^ While these findings demonstrate the short-term impact of recreational drug use on ICCU patients, the long-term impact, specifically in ACS patients and its subgroups, remains unclear. Therefore, this study investigated the 1-year prognostic impact of recreational drug use on outcomes in ICCU patients with ACS, focusing on differences between NSTEMI and STEMI presentations.

## Methods

### Study population

This multicenter, prospective, observational study is part of the ADDICT-ICCU study, which included all consecutive patients aged ≥18 years admitted to ICCUs over 2 weeks in April 2021 at 39 centers across France, representing all administrative regions of the country (Supplemental Table 1). The study design has been previously detailed and published.^11^ This study specifically focused on patients diagnosed with ACS from the ADDICT-ICCU cohort, excluding those lost to follow-up or who died during hospitalization before ICCU discharge. Baseline characteristics were collected as described in Supplemental Method 1. The main admission diagnosis was adjudicated by 2 independent experts at discharge, following each center's guidelines (Supplemental Method 2). The primary exclusion criteria for the ADDICT-ICCU study were hospitalization for a planned interventional procedure or more than 24 hours at another facility before ICCU admission, thereby minimizing the risk of false-negative urine drug assays for recreational drug use. Patient management was determined by treating physicians in accordance with the European Society of Cardiology guidelines. The study was registered at ClinicalTrials.gov (National Clinical Trial 05,063,097) and approved by the Committee for the Protection of Human Subjects, Ile de France-7, France (Assistance Publique-Hôpitaux de Paris190870). All participants provided written informed consent, and no patients were involved in the study design process.

### Assessment of drug detection

The following recreational drugs were evaluated for all consecutive patients by urine drug assay using a cartridge-based system (NarcoCheck, Kappa City Biotech Société par Actions Simplifiée, Montluçon, France) as soon as possible, at most within 2 hours of admission to the ICCU: 1) cannabinoids (tetrahydrocannabinol), including cannabis and hashish; 2) cocaine and metabolites, including crack; 3) amphetamines; 4) 3,4-Methylenedioxymethamphetamine; and 5) heroin and other opioids (Supplemental Figure 1). Details regarding the reliability of the NarcoCheck, previously published, are provided in the Supplemental Table 2.^12^ Importantly, urine tests for opioids were deemed negative for patients who had received morphine or other opioids for pain management during the initial management before ICCU admission.

### Patient follow-up and clinical outcomes

The follow-up process began following hospital discharge and involved either routine clinical visits or direct communication with the patient and their referring cardiologist (Supplemental Table 3). All patients were prospectively followed up to 1 year. Data collection concluded by June 2022. The primary composite outcome was the occurrence of at least one MACE, defined as cardiovascular death, nonfatal myocardial infarction, or stroke. Cardiovascular death included sudden cardiac death with documented fatal arrhythmias or death immediately preceded by acute myocardial infarction, acute heart failure exacerbation, or stroke. Nonfatal myocardial infarction was defined as typical angina of ≥20 minutes duration, electrocardiogram changes, and a rise in troponin or creatine kinase level exceeding the 99th percentile of the upper reference limit.^13^ Stroke diagnoses required imaging confirmation using cerebral magnetic resonance or computed tomography. All clinical events were defined according to the published standardized definitions.^14^ Adjudication of clinical events was performed by an independent committee of 2 senior cardiologists who reviewed anonymized medical records and reached a consensus. For event-free survival analysis, only the first MACE event per patient was included in the analysis.

### Statistical analysis

Patient characteristics are summarized as mean ± standard deviation for normally distributed data, and as median with interquartile range for nonnormally distributed data, with normality assessed through graphical methods for normality. Categorical variables are summarized with counts and proportions. Group comparisons for quantitative and qualitative variables were carried out with the Student’s *t*-test, Mann-Whitney test, or Pearson’s Chi-squared test, depending on the statistical distribution of the variables. Follow-up data are presented as median with interquartile range.

MACE-free survival curves were estimated using the Kaplan-Meier method, and differences between groups were compared using the log-rank test. Furthermore, clinical outcomes were analyzed using multivariable models, with covariates selected using 2 different approaches. The first model included prognostic variables identified via data-driven least absolute shrinkage and selection operator (LASSO) regression analysis, which facilitated the selection of the most statistically relevant predictors while excluding the less-relevant predictors. The second model included traditional prognostic variables known to influence MACE outcomes. To limit the number of covariates in each model and avoid overfitting given the sample size, 2 multivariable Cox proportional hazards models were performed for the overall ACS population to identify predictors of MACE among ACS patients with or without recreational drug use at 1-year follow-up.

1. Model 1 included variables selected by LASSO regression: age, drug detection, principal cardiac diagnosis (NSTEMI or STEMI) at admission, congestive heart failure signs, creatininemia, and Killip class.
2. Model 2 included traditional prognostic variables: age, sex, principal cardiac diagnosis at admission, diabetes, drug detection, and smoking.

This method of using 2 multivariable Cox proportional hazards models was applied to the analyses of the subpopulations of STEMI and NSTEMI.

To validate the main logistic regression analysis in the STEMI and NSTEMI subpopulations, an additional analysis was conducted using propensity score matching, with principal cardiac diagnosis as the main matching variable. Propensity scores were generated using a logistic regression model to balance baseline characteristics between patients with STEMI vs NSTEMI.^15^ To minimize potential selection bias, the impact of recreational drugs use on MACE at 1-year follow-up was assessed using a 1:1 propensity score–matched population (with vs without recreational drugs detected, R software package “MatchIt,” version 3.0.2). The probit model with 1-to-1 nearest neighbor matching and without replacement was used to identify one patient without recreational drugs detected for every patient with recreational drugs detected. Variables used to calculate the propensity score included baseline characteristics and the main admission diagnosis. Imbalances between groups were assessed using absolute standardized mean differences calculated using Yang and Dalton’s method, with a threshold of <0.2 indicating covariate balance (Supplemental Method 3).^16^ Prespecified subgroup analyses were performed according to the main admission diagnosis and recreational drugs detected. A two-tailed *P* value < 0.05 was considered statistically significant. All analyses were conducted using R software, version 3.6.3 (R Project for Statistical Computing, R Foundation).

## Results

### Study population

**Figure 1:**
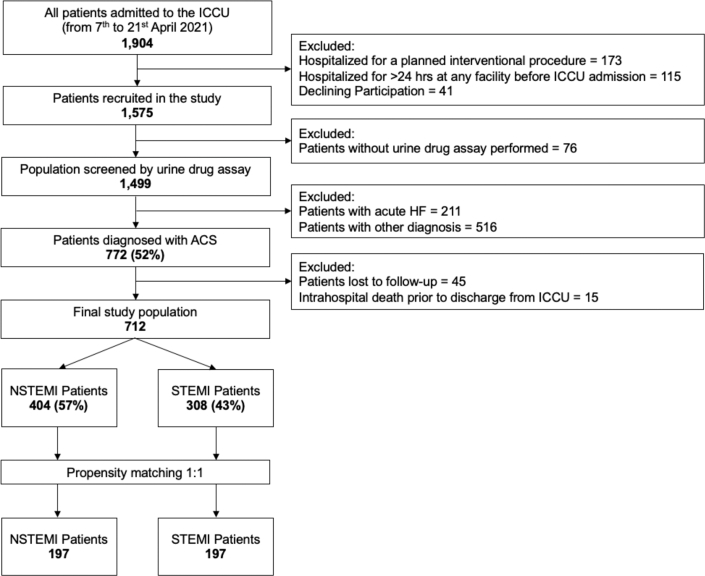
**Flowchart of the Study Population** This flow diagram outlines the selection process of patients admitted to ICCUs between April 7 and April 21, 2021. Of the 1,904 patients initially admitted, 1,575 were recruited. Following exclusion of those without urine drug screening or with diagnoses other than ACS, 712 patients remained in the final study cohort. Among these, 404 were diagnosed with NSTEMI, and 308 with STEMI. A 1:1 propensity score matching was subsequently performed, resulting in matched cohorts of 197 NSTEMI and 197 STEMI patients. ACS = acute coronary syndrome; HF = heart failure; ICCU = intensive cardiac care unit; NSTEMI = non-ST-segment elevation myocardial infarction; STEMI = ST-segment elevation myocardial infarction.

Between April 7 and 21, 2021, 1,904 patients were admitted to ICCUs across 39 participating centers in France. From this initial group of patients, 1,575 were enrolled in the ADDICT-ICCU study after excluding those with planned interventional procedures (N = 173), hospitalization exceeding 24 hours before ICCU admission (N = 115), or who declined participation (N = 41). Among the enrolled patients, 1,499 underwent drug screening using urine drug assay and constituted the primary cohort for the ADDICT-ICCU study. Across the primary population, 51.5% (N = 772) of patients were diagnosed with ACS. After excluding patients lost to follow-up (N = 45) or who died in hospital before discharge (N = 15), the final study population included 712 ACS patients. This cohort was used to evaluate the prognostic impact of recreational drug use on major adverse cardiovascular events in ACS patients discharged from the ICCU at 1-year follow-up (Figure 1).

### Impact of recreational drug use on the total population of ACS patients

In the total population of patients with ACS (N = 712), the mean age was 64 ± 13 years; 74.6% (N = 531) were males; 13.5% (N = 96) tested positive for drug detection; 56.7% (N = 404) presented with NSTEMI; and 43.3% (N = 308) presented with STEMI (Table 1). Among the 96 drug-positive patients, opioids were detected in 34, stimulants in 18, depressants in 8, and tetrahydrocannabinol in 86, with some patients testing positive for multiple substances. At the 1-year follow-up, 7.0% (N = 50) of patients experienced MACE defined as a stroke, nonfatal myocardial infarction, or cardiovascular death. Among those who experienced MACE, 76% (N = 38) tested negative for recreational drug use, representing 6.2% of the overall drug-negative group (N = 616). Conversely, 24% (N = 12) of patients with MACE tested positive for recreational drug use, corresponding to 12.5% of the drug-positive group (N = 96) (Supplemental Tables 4 and 5).

**Table 1:** Baseline Characteristics of the ACS Population and NSTEMI and STEMI Subpopulations According to Recreational Drug Detection

	Overall ACS Population (N = 712)	NSTEMI Population (N = 404)			STEMI Population (N = 308)		
		No Recreational Drug Detected (n = 349)	Recreational Drug Detected (n = 55)	*P* Value	No Recreational Drug Detected (n = 267)	Recreational Drug Detected (n = 41)	*P* Value
Age, y	64 ± 13	66 ± 12	58 ± 12	**<0.001**	64 ± 12	49 ± 12	**<0.001**
Men, n (%)	531 (74.6)	245 (70.2)	44 (80.0)	0.134	204 (76.4)	38 (92.7)	**0.018**
Body mass index, kg/m^2^	27.3 ± 5.2	27.6 ± 5.4	26.2 ± 5.6	**0.049**	27.2 ± 4.9	26.1 ± 4.5	0.119
CV risk factors, n (%)							
Hypertension	394 (55.3)	230 (65.9)	23 (41.8)	**<0.001**	131 (49.1)	10 (24.4)	**0.003**
Diabetes	162 (22.8)	101 (28.9)	6 (10.9)	**0.005**	51 (19.1)	4 (9.8)	0.146
Dyslipidemia	286 (40.2)	166 (47.6)	21 (38.2)	0.195	86 (32.2)	13 (31.7)	0.949
Family history of CAD	151 (21.2)	67 (19.2)	16 (29.1)	0.091	60 (22.5)	8 (19.5)	0.671
Smoking	231 (32.4)	75 (21.5)	31 (56.4)	**<0.001**	93 (34.8)	32 (78.0)	**<0.001**
Medical history of CV disease, n (%)							
History of ACS	110 (15.4)	68 (19.5)	10 (18.2)	0.820	27 (10.1)	5 (12.2)	0.594
Previous PCI	429 (60.3)	171 (49.0)	29 (52.7)	0.607	197 (73.8)	32 (78.0)	0.560
History of coronary disease^a^	491 (69.0)	216 (61.9)	33 (60.0)	0.789	208 (77.9)	34 (82.9)	0.465
History of CKD^b^	51 (7.2)	36 (10.3)	4 (7.3)	0.483	11 (4.1)	0 (0.0)	0.371
History of CV disease^c^	516 (72.5)	235 (67.3)	34 (61.8)	0.420	213 (79.8)	34 (82.9)	0.637
Alcohol consumption	403 (57.6)	189 (54.8)	31 (57.4)	0.718	156 (60.0)	27 (65.9)	0.476
Clinical data on admission							
Systolic blood pressure, mm Hg	136 ± 26	144 ± 25	137 ± 26	**0.043**	128 ± 23	124 ± 20	0.256
Heart rate, beats/min	79 ± 18	77 ± 17	86 ± 21	**0.002**	79 ± 18	85 ± 21	0.064
Oxygen saturation, %	97.3 ± 4.4	97.3 ± 2.5	95.7 ± 13.4	0.603	97.4 ± 2.5	98.0 ± 1.8	0.180
ICCU hospitalization duration, days	6.9 ± 15.8	6.8 ± 16.0	6.1 ± 5.1	0.460	7.1 ± 18.0	6.4 ± 4.6	0.420
Killip class				0.796			>0.999
1	649 (91.3)	320 (91.7)	50 (90.9)		241 (90.6)	38 (92.7)	
≥2	62 (8.7)	29 (8.3)	5 (9.1)		25 (9.4)	3 (7.3)	
CHF signs	123 (17.3)	59 (16.9)	12 (21.8)	0.374	45 (16.9)	7 (17.1)	0.972
Laboratory results							
Hemoglobin, g/dL	13.9 ± 1.8	13.7 ± 1.8	14.3 ± 1.8	**0.008**	14.0 ± 1.7	14.6 ± 1.6	0.096
Creatininemia, μmol/L	91 ± 64	97 ± 75	81 ± 27	0.268	86 ± 58	84 ± 29	0.316
High-sensitivity cardiac troponin peak^d^	638 ± 1,523	149 ± 398	224 ± 622	0.279	1,286 ± 2,207	1,230 ± 1,374	0.243
BNP, pg/mL	289 ± 613	385 ± 741	192 ± 306	0.227	207 ± 475	106 ± 173	0.347
Echocardiography data							
LV ejection fraction, %	53 ± 11	55 ± 11	54 ± 10	0.291	50 ± 10	49 ± 11	0.338
TAPSE, mm	21.5 ± 4.1	21.7 ± 4.0	21.3 ± 5.5	0.599	21.5 ± 3.9	19.8 ± 3.4	**0.017**
sPAP, mm Hg	32 ± 12	30 ± 11	40 ± 13	**<0.001**	29 ± 11	38 ± 12	**<0.001**
TAPSE/sPAP ratio	0.8 ± 0.4	0.8 ± 0.4	0.6 ± 0.3	**<0.001**	0.8 ± 0.4	0.6 ± 0.2	**<0.001**
VTI, cm	19.4 ± 4.8	20.3 ± 5.2	18.7 ± 5.1	**0.015**	18.8 ± 4.0	17.3 ± 4.3	0.065
E/e' ratio	8.3 ± 3.0	8.4 ± 2.9	8.7 ± 3.5	0.733	8.4 ± 3.0	7.6 ± 3.4	**0.035**
E/A ratio	1.0 ± 0.5	1.0 ± 0.5	1.2 ± 0.5	**0.003**	1.0 ± 0.4	1.1 ± 0.5	0.123

Values are n (%), mean ± SD, or median (IQR).

**Bold** values indicate the total number of patients at each stage of the study.

ACS = acute coronary syndrome; BNP = B-type natriuretic peptide; CABG = coronary artery bypass graft; CAD = coronary artery disease; CHF = congestive heart failure; CKD = chronic kidney disease; CV = cardiovascular; ICCU = intensive cardiac care unit; ICD = implantable cardioverter-defibrillator; LV = left ventricle; MI = myocardial infarction; NSTEMI = non-ST-segment elevation myocardial infarction; PCI = percutaneous coronary intervention; SD = standard deviation; sPAP = systolic pulmonary artery pressure; STEMI = ST-segment elevation myocardial infarction; TAPSE = tricuspid annular plane systolic excursion; VTI = velocity time integral.

a History of coronary disease defined by the presence of ≥1 of the following: ACS, angioplasty, or coronary bypass.

b CKD defined by history of chronic kidney disease with glomerular filtration rate <60 mL/min/m^2^.

c CV disease defined by the presence of known MI, previous PCI, previous CABG, peripheral atheroma with revascularization, stroke, history of heart failure, history of atrial fibrillation, history of surgery for valvular heart disease, pacemaker or ICD, and cardiomyopathies.

d Represents the n-fold troponin levels.

In the univariable analysis (Table 2), the detection of recreational drugs was associated with an increased risk of MACE at 1-year follow-up (HR: 2.08, 95% CI: 1.09-3.98, *P* = 0.027). The multivariable analysis demonstrated that the detection of recreational drugs was independently associated with a significantly higher incidence and risk of MACE in ACS patients discharged from the ICCUs at 1-year follow-up (adjusted HR: 2.70, 95% CI: 1.30-5.57, *P* = 0.013; model 1: covariates selected via LASSO regression). This association was also significant after adjustment for known prognostic predictors of MACE (model 2: adjusted HR: 3.01, 95% CI: 1.46-6.21, *P* = 0.006) (Supplemental Table 6).

**Table 2:** Univariable and Multivariable Analyses of Recreational Drug Use for MACE in ACS Patients (N = 712) According to LASSO Determinants

	Univariable		Multivariable^a^	
	HR (95% CI)	*P* Value	HR (95% CI)	*P* Value
Age, y	1.02 (1.00-1.04)	0.118	1.03 (1.00-1.05)	**0.046**
Men, n (%)	1.22 (0.62-2.38)	0.563	-	-
Body mass index, kg/m^2^	0.95 (0.90-1.02)	0.142	-	-
Admission diagnosis				
NSTEMI	-	-	-	-
STEMI	1.85 (1.05-3.24)	**0.032**	2.02 (1.14-3.59)	**0.015**
CV risk factors, n (%)				
Hypertension	0.87 (1.07-1.66)	0.635	-	-
Diabetes	1.21 (0.64-2.27)	0.559	-	-
Dyslipidemia	1.29 (0.74-2.25)	0.372	-	-
Family history of CAD	1.04 (0.53-2.04)	0.900	-	-
Smoking	1.07 (0.60-1.92)	0.819	-	-
Medical history of CV disease, n (%)			-	-
History of ACS	1.05 (0.49-2.24)	0.894	-	-
Previous PCI	0.99 (0.56-1.75)	0.984	-	-
History of coronary disease^b^	1.07 (0.58-1.95)	0.833	-	-
History of CKD^c^	1.48 (0.59-3.72)	0.408	-	-
History of CV disease^d^	0.99 (0.53-1.83)	0.969	-	-
Alcohol consumption	0.64 (0.37-1.13)	0.124	-	-
Clinical data on admission				
Systolic blood pressure, mm Hg	1.00 (0.99-1.01)	0.536	-	-
Heart rate, beats/min	1.00 (0.99-1.02)	0.654	-	-
Oxygen saturation, %	1.01 (0.93-1.10)	0.821	-	-
ICCU hospitalization duration, days	1.01 (1.00-1.02)	0.200	-	-
Killip class		**0.018**		0.47
1	-		-	
≥2	2.38 (1.16-4.90)		1.49 (0.49-4.50)	
CHF signs	2.09 (1.14-3.83)	**0.017**	1.17 (0.45-3.01)	0.75
Urine drug assay				
Drug detection	2.08 (1.09-3.98)	**0.027**	2.70 (1.30-5.57)	**0.013**
Cannabis	1.87 (0.93-3.73)	0.077	-	-
Stimulants^e^	1.60 (0.39-6.57)	0.517	-	-
Opioids^f^	4.81 (2.34-9.91)	**<0.001**	-	-
Depressants^g^	1.76 (0.24-12.7)	0.576	-	-
Laboratory results				
Hemoglobin, g/dL	0.95 (0.81-1.12)	0.558	-	-
Creatininemia, μmol/L	1.00 (1.00-1.01)	**0.014**	1.00 (1.00-1.01)	0.063
High-sensitivity cardiac troponin peak^h^	1.00 (1.00-1.00)	0.882	-	-
BNP, pg/mL	1.00 (1.00-1.00)	0.175		
Echocardiography data				
LV ejection fraction, %	0.98 (0.95-1.00)	**0.047**	-	-
TAPSE, mm	0.92 (0.86-0.99)	**0.023**	-	-
sPAP, mm Hg	1.03 (1.01-1.05)	**0.005**	-	-
TAPSE/sPAP ratio	0.10 (0.03-0.39)	**<0.001**	-	-
VTI, cm	0.97 (0.90-1.03)	0.314	-	-
E/e ratio	1.08 (0.99-1.19)	0.097	-	-
E/A ratio	1.26 (0.70-2.29)	0.446	-	-

**Bold** values indicate the 2-tailed *P*-value reached statistical significance (<0.05).

ACS = acute coronary syndrome; BNP = B-type natriuretic peptide; CABG = coronary artery bypass graft; CAD = coronary artery disease; CHF = congestive heart failure; CI = confidence interval; CKD = chronic kidney disease; CV = cardiovascular; EDDP = 2-Ethylidene-1,5-Dimethyl-3,3-Diphenylpyrrolidine; HR = hazard ratio; ICCU = intensive cardiac care unit; ICD = implantable cardioverter-defibrillator; Inf = infinity; LASSO = least absolute shrinkage and selection operator; LV = left ventricle; MACE = major adverse cardiovascular events; MDMA = 3,4-Methylenedioxymethamphetamine; MI = myocardial infarction; NSTEMI = non-ST-segment elevation myocardial infarction; PCI = percutaneous coronary intervention; sPAP = systolic pulmonary artery pressure; STEMI = ST-segment elevation myocardial infarction; TAPSE = tricuspid annular plane systolic excursion; VTI = velocity time integral.

a Covariates determined by LASSO regression: age, admission diagnosis, drug detected, Killip class, creatininemia, CHF diagnosis.

b History of coronary disease defined by the presence of ≥1 of the following: ACS, angioplasty, or coronary bypass.

c CKD defined by history of chronic kidney disease with glomerular filtration rate <60 mL/min/m^2^.

d CV disease defined by the presence of known MI, previous PCI, previous CABG, peripheral atheroma with revascularization, stroke, history of heart failure, history of atrial fibrillation, history of surgery for valvular heart disease, pacemaker or ICD, and cardiomyopathies.

e Stimulants tested for: cocaine, amphetamine, and MDMA.

f Opioids tested for: morphine, buprenorphine, and EDDP.

g Depressants tested for: benzodiazepine and barbiturate.

h Represents the n-fold troponin levels.

### Impact of recreational drug use in the subpopulations of STEMI and NSTEMI ACS patients

In the STEMI group (N = 308), the mean age was 62 ± 13 years; 78.6% (N = 242) were males; 13.3% (N = 41) tested positive for drug detection; and 9.4% (N = 29) experienced MACE at 1-year follow-up (Table 1). Among the patients who experienced MACE, 31.0% (N = 9) tested positive for drug detection, representing 22% of the drug-positive STEMI population (N = 41). Conversely, the remaining 69.0% (N = 20) of patients who experienced MACE and tested negative for recreational drug use represented 7.5% of the drug-negative STEMI population (N = 267) (Supplemental Table 7). In the NSTEMI group (N = 404), the mean age was 65 ± 12 years; 71.5% (N = 289) were males; 13.6% (N = 55) tested positive for drug detection; and 5.2% (N = 21) experienced MACE at 1-year follow-up (Supplemental Table 7). Among the patients who experienced MACE, 5.5% (N = 3) tested positive for drug detection, representing 5.5% of the drug-positive NSTEMI group (N = 55). Conversely, 94.5% (N = 18) of patients that experienced MACE and tested negative for recreational drug use represented 5.2% of the drug-negative NSTEMI population (N = 349) (Supplemental Table 7).

In the univariable analysis (Supplemental Table 8) of STEMI and NSTEMI ACS patients, recreational drug detection was associated with MACE in STEMI patients at 1-year follow-up (HR: 3.13, 95% CI: 1.42-6.87, *P* = 0.004). However, this association was not evident in NSTEMI patients (HR: 1.06, 95% CI: 0.31-3.59, *P* = 0.929). As summarized in the Central Illustration, the multivariable analysis demonstrated that the multivariable analysis demonstrated that the detection of recreational drugs was significantly associated with a higher incidence and risk of MACE in the STEMI group discharged from the ICCU at 1-year follow-up (adjusted HR: 4.11, 95% CI: 1.60-10.5, *P* = 0.005; covariates selected via LASSO regression) (Table 3). This association remained significant even after adjustment for known prognostic determinants of MACE (model 2: adjusted HR: 4.05, 95% CI: 1.60-10.3, *P* = 0.005) (Supplemental Table 9). However, this association was not evident in the NSTEMI group even after adjustment for covariates selected by LASSO regression (model 1: adjusted HR: 1.39, 95% CI: 0.40-4.87, *P* = 0.62) (Table 3) and for known predictors of MACE (model 2: adjusted HR: 1.61, 95% CI: 0.45-5.71, *P* = 0.48) (Supplemental Table 9).

**Central Illustration:**
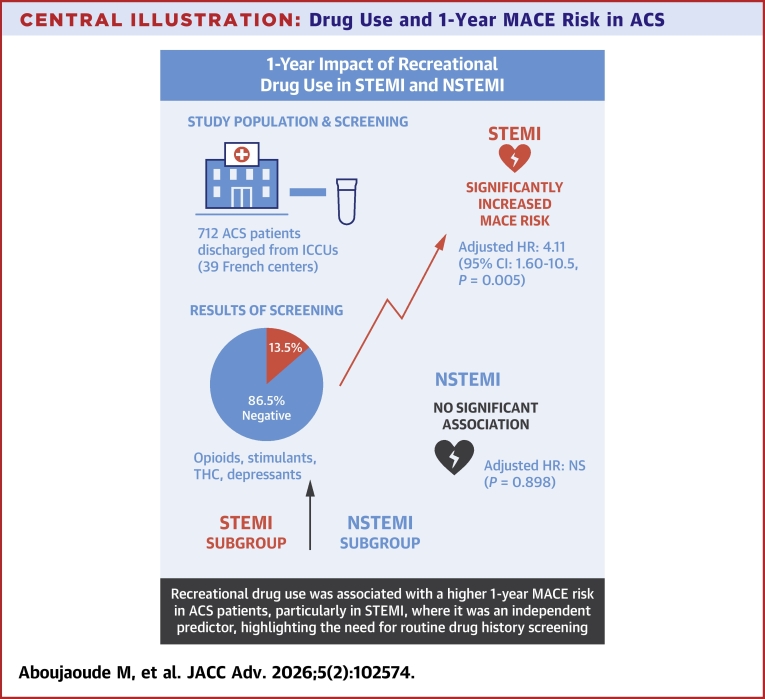
**Drug Use and 1-Year MACE Risk in ACS** This illustration summarizes the 1-year prognostic impact of recreational drug use in ACS patients from the ADDICT-ICCU multicenter cohort. Among 712 ACS patients systematically screened by urine drug assay at ICCU admission, 13.5% tested positive for recreational drugs. Drug-positive patients exhibited a higher 1-year rate of MACE, defined as cardiovascular death, nonfatal myocardial infarction, or stroke (12.5% vs 6.2%). In multivariable Cox analysis, recreational drug use independently predicted 1-year MACE in the overall ACS population and showed a pronounced effect in the STEMI subgroup, where drug use was associated with a near four-fold increased risk. No significant association was observed in NSTEMI. These findings support routine screening for recreational drug use at admission to identify ACS patients, particularly those with STEMI, at elevated long-term cardiovascular risk. Abbreviations: ACS = acute coronary syndrome; ADDICT-ICCU = Addiction in Intensive Cardiac Care Units; CI = confidence interval; HR = hazard ratio; ICCU = intensive cardiac care unit; MACE = major adverse cardiovascular events; NSTEMI = non-ST-segment elevation myocardial infarction; STEMI = ST-segment elevation myocardial infarction.

**Table 3:** Multivariable Analyses of Recreational Drug Detected for MACE in NSTEMI and STEMI Patients

	NSTEMI^a^ (N = 404)		STEMI^b^ (N = 308)	
	HR (95% CI)	*P* Value	HR (95% CI)	*P* Value
Age	1.04 (1.00-1.08)	**0.043**	1.02 (0.98-1.05)	0.33
Drug detected	1.39 (0.40-4.87)	0.62	4.11 (1.60-10.5)	**0.005**
BMI	-	-	0.90 (0.82-0.99)	**0.021**
CHF signs	-	-	1.89 (0.81-4.40)	0.15
Creatininemia	1.00 (1.00-1.01)	0.20	-	-
Killip score		0.085		-
1	-		-	
≥2	2.70 (0.95-7.65)		-	

**Bold** values indicate the 2-tailed *P*-value reached statistical significance (<0.05).

BMI = body mass index; CHF = congestive heart failure; LASSO = least absolute shrinkage and selection operator; MACE = major adverse cardiovascular events; NSTEMI = non-ST-segment elevation myocardial infarction; STEMI = ST-segment elevation myocardial infarction.

a Covariates determined by LASSO regression: age, drug detected, creatininemia, and Killip score.

b Covariates determined by LASSO regression: age, drug detected, BMI, and CHF signs.

To further substantiate these findings, a secondary analysis was performed using propensity score matching guided by a general linear model. This analysis included 394 patients, evenly divided between ACS patients with STEMI (N = 197) and NSTEMI (N = 197). Matching criteria included age, sex, drug detection, congestive heart failure signs, history of coronary artery disease, dyslipidemia, troponin levels, smoking status, prior percutaneous coronary intervention, history of ACS, diabetes, hypertension, and history of cardiovascular disease. The baseline characteristics of the propensity-matched population according to recreational drug use are presented in Supplemental Table 10. In this propensity-matched cohort, univariate analysis (Table 4) revealed that the detection of recreational drugs was significantly associated with a higher risk and incidence of MACE in the STEMI population (HR: 3.39, 95% CI: 1.19-9.62, *P* = 0.022). In contrast, no such association between recreational drug use and MACE was observed in the NSTEMI population (HR: 0.87, 95% CI: 0.11-7.08, *P* = 0.898).

**Table 4:** Univariable Analyses of Recreational Drug Use for MACE in STEMI and NSTEMI Patients After Propensity Score Matching for Cardiac Diagnosis

	NSTEMI (N = 197)		STEMI (N = 197)	
	HR (95% CI)	*P* Value	HR (95% CI)	*P* Value
Age, y	1.02 (0.96-1.08)	0.579	1.01 (0.97-1.05)	0.643
Men, n (%)	0.39 (0.09-1.65)	0.202	2.66 (0.61-11.6)	0.193
Body mass index, kg/m^2^	1.05 (0.94-1.19)	0.379	0.95 (0.85-1.06)	0.384
CV risk factors, n (%)				
Hypertension	1.93 (0.46-8.08)	0.367	0.80 (0.31-2.06)	0.640
Diabetes	1.39 (0.28-6.87)	0.689	1.18 (0.39-3.63)	0.770
Dyslipidemia	1.55 (0.37-6.47)	0.551	0.82 (0.29-2.33)	0.710
Family history of CAD	1.30 (0.26-6.44)	0.748	0.48 (0.11-2.10)	0.329
Smoking	0.96 (0.23-4.00)	0.951	1.32 (0.50-3.48)	0.568
Medical history of CV disease, n (%)				
History of ACS	1.14 (0.14-9.23)	0.905	1.14 (0.26-5.00)	0.859
Previous PCI	0.60 (0.14-2.50)	0.482	0.89 (0.33-2.40)	0.812
History of coronary disease^a^	0.90 (0.18-4.46)	0.897	0.67 (0.25-1.81)	0.429
History of CKD^b^	3.58 (0.72-17.7)	0.119	1.27 (0.17-9.60)	0.815
History of CV disease^c^	0.80 (0.16-3.95)	0.781	0.77 (0.27-2.18)	0.619
Alcohol consumption	0.58 (0.15-2.32)	0.442	0.37 (0.13-1.01)	0.052
Clinical data on admission				
Systolic blood pressure, mm Hg	1.01 (0.98-1.03)	0.714	0.98 (0.96-1.01)	0.141
Heart rate, beats/min	1.01 (0.97-1.04)	0.610	1.00 (0.98-1.03)	0.835
Oxygen saturation, %	1.14 (0.78-1.67)	0.501	1.02 (0.84-1.23)	0.849
ICCU hospitalization duration, days	0.91 (0.70-1.18)	0.477	1.01 (1.00-1.02)	0.058
Killip class		0.121		0.152
1	-		-	
≥2	3.55 (0.72-17.6)		2.49 (0.72-8.66)	
CHF signs	1.77 (0.36-8.78)	0.483	2.93 (1.08-7.93)	**0.034**
Urine drug assay				
Drug detection	0.87 (0.11-7.08)	0.898	3.39 (1.19-9.62)	**0.022**
Cannabis	0.87 (0.11-7.08)	0.898	3.80 (1.34-10.8)	**0.012**
Stimulants^d^	Inf^e^	0.999	Inf^e^	0.998
Opioids^f^	5.13 (0.63-41.7)	0.126	6.19 (2.01-19.0)	**0.001**
Depressants^g^	Inf^e^	0.998	4.21 (0.56-31.8)	0.163
Laboratory results				
Hemoglobin, g/dL	0.82 (0.56-1.18)	0.282	1.00 (0.75-1.32)	0.973
Creatininemia, μmol/L	1.00 (0.99-1.01)	0.839	1.00 (1.00-1.01)	**0.017**
High-sensitivity cardiac troponin peak^h^	1.00 (1.00-1.00)	0.674	1.00 (1.00-1.00)	0.643
BNP, pg/mL	1.00 (1.00-1.00)	0.640	1.00 (1.00-1.00)	0.987
Echocardiography data				
LV ejection fraction, %	0.96 (0.91-1.02)	0.172	0.98 (0.94-1.03)	0.469
TAPSE, mm	0.95 (0.80-1.13)	0.569	0.89 (0.78-1.02)	0.092
sPAP, mm Hg	1.04 (0.98-1.09)	0.191	1.03 (0.99-1.07)	0.116
TAPSE/sPAP ratio	0.18 (0.01-3.32)	0.246	0.06 (0.01-0.65)	**0.021**
VTI, cm	1.01 (0.86-1.18)	0.947	0.95 (0.84-1.07)	0.380
E/e ratio	1.15 (0.87-1.51)	0.327	1.15 (0.98-1.36)	0.091
E/A ratio	2.13 (0.51-8.91)	0.301	1.24 (0.40-3.82)	0.707

**Bold** values indicate the 2-tailed *P*-value reached statistical significance (<0.05).

ACS = acute coronary syndrome; BNP = B-type natriuretic peptide; CABG = coronary artery bypass graft; CAD = coronary artery disease; CHF = congestive heart failure; CI = confidence interval; CKD = chronic kidney disease; CV = cardiovascular; EDDP = 2-ethylidene-1,5-dimethyl-3,3-diphenylpyrrolidine; HR = hazard ratio; ICCU = intensive cardiac care unit; ICD = implantable cardioverter-defibrillator; Inf = infinity; LV = left ventricle; NSTEMI = non-ST-segment elevation myocardial infarction; MACE = major adverse cardiovascular events; MDMA = 3,4-methylenedioxymethamphetamine; MI = myocardial infarction; PCI = percutaneous coronary intervention; sPAP = systolic pulmonary artery pressure; STEMI = ST-segment elevation myocardial infarction; TAPSE = tricuspid annular plane systolic excursion; VTI = velocity time integral.

a History of coronary disease defined by the presence of ≥1 of the following: ACS, angioplasty, or coronary bypass.

b CKD defined by history of chronic kidney disease with glomerular filtration rate <60 mL/min/m^2^.

c CV disease defined by the presence of known MI, previous PCI, previous CABG, peripheral atheroma with revascularization, stroke, history of heart failure, history of atrial fibrillation, history of surgery for valvular heart disease, pacemaker or ICD, and cardiomyopathies.

d Stimulants tested for: cocaine, amphetamine, and MDMA.

e Nonestimable hazard ratio due to zero outcome events in the exposed group, resulting in an infinite confidence interval.

f Opioids tested for: morphine, buprenorphine, and EDDP.

g Depressants tested for: benzodiazepine and barbiturate.

h Represents the n-fold troponin levels.

## Discussion

In this prospective multicenter cohort study, consecutive patients admitted to ICCUs across France for acute cardiovascular events and diagnosed with ACS were systematically tested for recreational drug use using urine drug assay. The main findings were as follows: 1) the detection of recreational drugs was a strong independent predictor of MACE at 1-year follow-up in the total ACS population; 2) this association was particularly pronounced among STEMI patients, whereas NSTEMI patients exhibited no such association between recreational drug use and MACE.

### Association between recreational drug detection and MACE in ACS patients

**Figure 2:**
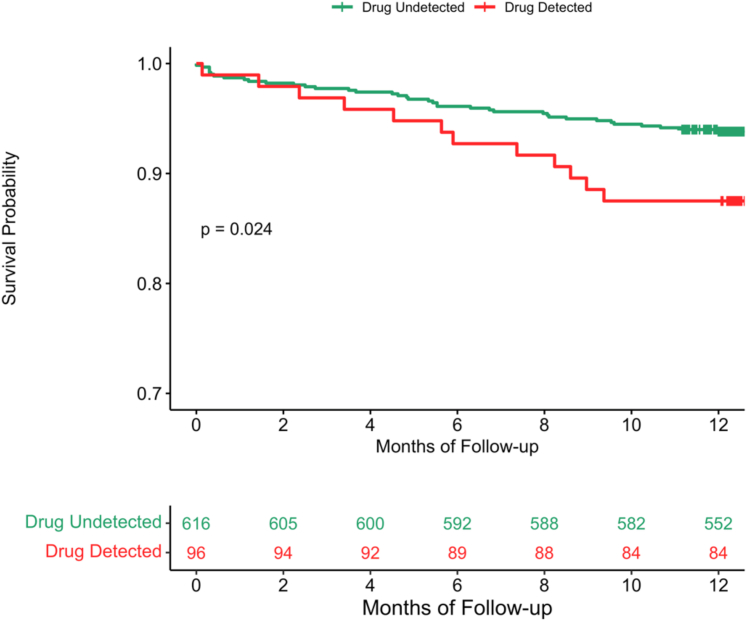
**MACE-Free Survival by Recreational Drug Use (N = 712)** This Kaplan-Meier curve illustrates 1-year survival without MACE in patients diagnosed with ACS, stratified by recreational drug detection at admission. Patients with positive drug detection (red line) demonstrated significantly lower MACE-free survival than those with negative drug detection (green line) (*P* = 0.024). The difference in survival probability progressively increased over the follow-up period. These findings suggest that recreational drug use at presentation is an independent predictor of adverse cardiovascular outcomes in ACS patients. ACS = acute coronary syndrome; MACE = major adverse cardiovascular events.

Our research team previously demonstrated that recent recreational drug use was independently associated with increased in-hospital adverse events among ICCU patients admitted for acute cardiovascular events.^9^ Several other studies have corroborated these results in ACS patients, showing that short-term recreational drug use is linked with worse outcomes.^5^^,^^17^^,^^18^ Gresnigt et al found that nearly one in five ACS patients under 50 years of age had recently used recreational drugs, with users more likely to present with STEMI and experience higher rates of ventricular arrhythmias and cardiogenic shock.^7^ However, these studies are largely retrospective and limited to in-hospital outcomes. To our knowledge, our study is the first prospective multicenter study to demonstrate a significant, independent association between recreational drugs use and MACE at 1-year follow-up in ICCU patients with ACS. Urine drug detection was significantly correlated with an increased incidence of stroke, nonfatal myocardial infarction, and cardiovascular death at 1-year follow-up, even after adjusting for known prognostic factors (Figure 2). Multivariable Cox analysis confirmed that recreational drug use was an independent predictor of MACE, revealing a 2.7-fold increase in risk at 1-year follow-up. Our findings establish recreational drug use as a clinically meaningful prognostic factor in ACS and underscore the importance of incorporating drug use history into risk assessment. Furthermore, these results highlight the potential long-term effects of drug use indicating a need for continued monitoring of recreational drug use within at least 1 year following discharge from the ICCU. While guidelines recommend declarative surveys for assessing recreational drug use among patients, the absence of systematic drug testing limits the accuracy of prevalence estimates and outcome predictions.^19^ Given this discrepancy, using urine screenings upon admittance to determine acute drug use enables identifying high-risk patients who may benefit from heightened monitoring and preventative interventions.

### Differential impact of recreational drug use on STEMI vs NSTEMI patients

**Figure 3:**
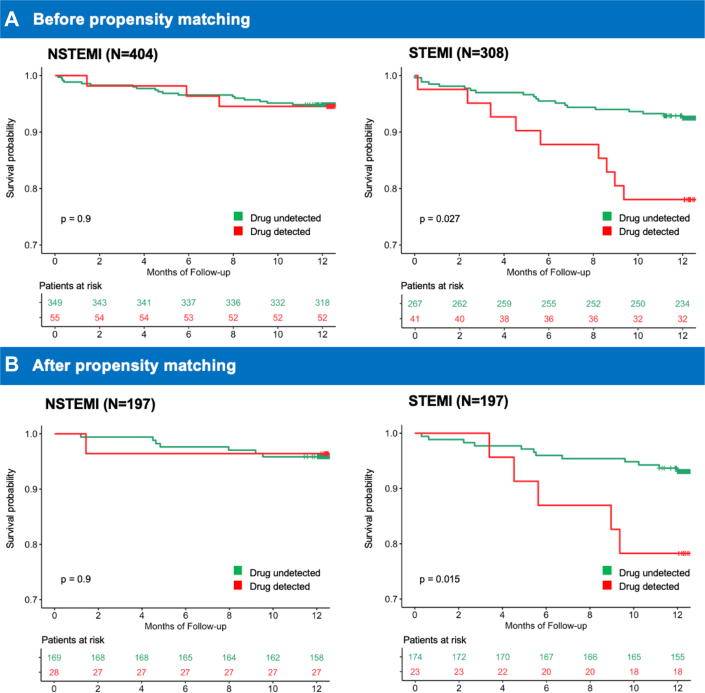
**MACE in STEMI vs NSTEMI Before and After Matching** The Kaplan–Meier survival curves depict 1-year survival without MACE stratified by recreational drug use detection in STEMI and NSTEMI patients. (A) Before propensity score matching, recreational drug use was associated with a significantly higher risk of MACE in STEMI patients (*P* = 0.027), but not in NSTEMI (*P* = 0.90). (B) After 1:1 propensity score matching, the difference in MACE risk remained significant in STEMI patients (*P* = 0.015), while NSTEMI showed no significant difference (*P* = 0.90). MACE = major adverse cardiovascular events; NSTEMI = non-ST-segment elevation myocardial infarction; STEMI = ST-segment elevation myocardial infarction.

Our prior research from the ADDICT-ICCU study found that recreational drug use was detected in 12.6% of STEMI patients and was independently associated with a 13-fold increase in in-hospital MACE, including cardiogenic shock, resuscitated cardiac arrest, and death.^10^ However, whether recreational drug use has different long-term prognostic implications in STEMI vs NSTEMI patients had remained unexplored. A key finding of this study sheds light on this question by demonstrating that recreational drug use was significantly associated with MACE at 1-year follow-up in STEMI patients, with at least a 4-fold increase in risk after adjusting for covariates in both multivariable Cox and propensity score-matched analysis. Conversely, drug use was not significantly associated with MACE in NSTEMI patients at 1-year follow-up (Figure 3). This differential association may be attributed to the distinct pathophysiology between STEMI and NSTEMI, including varying degrees of coronary plaque rupture, infarct extent, and electrocardiogram patterns. STEMI is characterized by complete coronary occlusion, transmural infarction, and electrocardiogram findings such as “tombstone” ST-elevation and pathologic Q-waves.^20^^,^^21^ Recreational drugs exert sympathomimetic effects, including increased heart rate, vasoconstriction, and heightened prothrombotic activity.^17^^,^^22^ These effects may amplify the ischemic burden in STEMI, contributing to worse long-term outcomes.^23^ These findings highlight the need for tailored risk-stratification approaches based on ACS subtype. While recreational drug use should be considered a potential risk factor for all ACS patients, the particularly strong association in STEMI patients suggests that this subgroup may benefit most from targeted interventions.

### Study Limitations

First, drug detection in the ICCU could not differentiate whether a positive drug-detection result reflected acute or chronic drug use. Although the urine drug assay employed detects drug use within approximately 2 to 6 days of consumption, it does not distinguish between chronic and acute drug use or their respective impacts on outcomes. Second, drug assay tests were only administered once at the beginning of the study, without further testing throughout the 1-year follow-up period. As a result, recreational drug use following discharge from the ICCU could have gone undetected and may have had a compounding effect on MACE at 1-year follow-up. Since no data were collected on the dose or frequency of drug use, some patients may have discontinued use after discharge. Despite this limitation, the persistent separation of Kaplan-Meier curves over 1 year suggests that baseline drug detection identifies patients at higher long-term risk. Third, while the study cohort size was sufficient to generate a significantly accurate prognostic relationship between drug detection and MACE, a larger sample size would have provided more power to the findings. This limitation was particularly relevant for the stimulant subgroup. In addition, a larger sample size would have allowed the exploration of the effects of multiple drug use contrasted to single drug use. Fourth, clinicians were aware of the urine drug assay results, which may have influenced medical management decisions, although the extent of this impact remains unclear. Lastly, as this study was centered in France, these findings may not be fully generalizable beyond France and Europe, particularly in regions where drug use patterns and other prognostic risk factors differ substantially.

## Conclusions

In this prospective multicenter observational study of patients admitted to ICCUs and diagnosed with ACS, recreational drug use was associated with a significantly higher risk of MACE at 1-year follow-up. This association was especially prominent in STEMI patients, where drug use was an independent predictor of MACE, whereas no such association was observed in NSTEMI patients. These findings underscore the importance of accurate drug use history in the risk stratification and management of ACS patients, especially those with STEMI.

## Funding support and author disclosures

Grant given by “Fondation Coeur et Recherche” (grant 01, 2017). The authors have reported that they have no relationships relevant to the contents of this paper to disclose.
