# Supplementary material for: Prognostic Impact of Recreational Drug Use on 1-Year Outcomes in NSTEMI and STEMI Patients
Source: JACC Adv. 2026 Jan 23;5(2):102574. doi: 10.1016/j.jacadv.2025.102574 (PMC12860991; doi:10.1016/j.jacadv.2025.102574)
Supplement: Supplemental Material [file mmc1.pdf]

<u>Supplemental Appendix</u>

Methods:

- Method 1: Baseline and in-hospital characteristics collection.
- Method 2: Definition of main admission diagnosis.
- Method 3: Absolute standardized mean differences in the propensity matched population.
- Method 4: Reasons for failure to perform drug urine assay.

Tables:

- Table 1: Detailed list of participating centers.
- Table 2: Reliability parameters of the urine drug assay.
- Table 3: List of ADDICT-ICCU investigators.
- Table 4: Baseline characteristics of the overall ACS population according to recreational drug detection.
- Table 5: Baseline characteristics of the overall ACS population according to MACE.
- Table 6: Univariable and multivariable analyses of recreational drug use for MACE in the ACS population (N=712) according to traditional prognostic factors.
- Table 7: Baseline characteristics of the ACS population and the NSTEMI and STEMI subpopulations according to MACE.
- Table 8: Univariable analyses of recreational drug detected for MACE in NSTEMI and STEMI patients before propensity score matching.
- Table 9: Multivariable analyses of recreational drug detected for MACE in NSTEMI and STEMI patients according to traditional prognostic factors.

- Table 10: Baseline characteristics of the ACS population and the propensity-matched subpopulation of NSTEMI and STEMI patients according to recreational drug detection.

Figures:

- Figure 1: Presentation of the urine drug assay.

References

This supplementary material has been provided by the authors to give readers additional information about their work.

Methods

Method 1. Baseline and in-hospital characteristics collection

Baseline characteristics

- Baseline data included the date of birth, gender, height, weight, temperature, systolic and diastolic blood pressures, heart rate, Glasgow score, Killip class, oxygen saturation and ventilation mode, presence of symptoms, list of medications at admission, history of cardiovascular disease, psychiatric illness (including major depressive disorder, bipolar disorder or schizophrenic disorder) or other significant clinical histories and main admission diagnosis.
- Regarding the drug addiction surveys, we assessed: i) declaration of psychoactive drug use (cannabinoids, cocaine, amphetamines, MDMA, heroin or other opioids); ii) smoking history (daily or nondaily smoker, cigarettes smoked per day by daily smokers, age started smoking, years smoked and e-cigarette use); iii) declaration of alcohol use.
- Of note, history of cardiovascular disease (CVD) was defined by the presence of: known MI, previous PCI, previous CABG, peripheral atheroma with revascularization, stroke, history of heart failure, history of atrial fibrillation, history of surgery for valvular heart disease, pacemaker or ICD, and cardiomyopathies.

Hospital clinical characteristics

- Electrocardiogram (ECG) and transthoracic echocardiography (TTE) with left ventricular ejection fraction (LVEF) were performed systematically within the first 24 hours of admission for all patients. Need for revascularization [percutaneous coronary intervention (PCI) or coronary artery bypass surgery (CABG)], ventricular arrhythmia (sustained ventricular tachycardia or fibrillation), cardiogenic shock, need for hemodynamic support, and urgent repeat revascularization were recorded.

- Laboratory results were also collected systematically upon admission, including hemoglobin, potassium, creatinine, the maximum peak of troponin (hsTNI), the Nterminal prohormone of B-type natriuretic peptide (NT-proBNP) or B-type natriuretic peptide (BNP).
- All the diagnostic procedures of cardiovascular imaging or invasive angiography reports were collected. All treatments introduced during hospitalization and the procedures performed were collected.
- The COVID status of each patient was systematically assessed at ICCU admission using RT-PCR, following current World Health Organization guidelines

Method 2. Definition of main admission diagnosis

- The medical reasons for admission and the main admission diagnosis were adjudicated by a committee of two experts at the end of the hospitalization in each center.
- Main admission diagnosis was categorized into different subgroups: i) ST elevation myocardial infarction (STEMI), including mechanical complication of acute coronary syndrome; ii) non-ST elevation myocardial infarction (NSTEMI); iii) acute heart failure; iv) myocarditis; v) pericarditis; vi) pulmonary embolism; vii) atrial arrhythmia; viii) ventricular arrhythmia; ix) cardiac conduction abnormalities; x) coronary spasm; xi) Takotsubo; xii) aortic dissection; xiii) spontaneous coronary dissection, xiv) chest pain without identified cardiac cause, and xv) other cardiovascular or non-cardiovascular diagnosis.
- Of note, other cardiovascular diagnoses included: infectious endocarditis, acute hypertensive crisis without heart failure, prosthetic valve dysfunction without heart failure, vagal discomfort or orthostatic hypotension without severe cardiac conduction abnormality detected, and monitoring after electrocution.
- Of note, other non-cardiovascular diagnoses included: gastric ulcer, pancreatitis, acute cholecystitis, anemia, exacerbation of COPD, severe asthma, lung or systemic infection, severe COVID-19, migraine crisis, palpitations or dyspnea without a cardiovascular diagnosis.

Main admission diagnosis	Definition
**Acute coronary syndrome**	Acute coronary syndrome will be defined by typical angina of ^{3} 20 min duration, ECG changes, and a rise in troponin or creatine kinase level above the 99th percentile of the upper reference limit after elimination of the differential diagnosis (myopericarditis, Takotsubo syndrome, Tachyarrhythmias, acute heart failure…).[1] Acute coronary syndrome will be classified as ST-segment elevation and non-ST-segment elevation categories.[2]
**Acute heart failure**	A hospitalization for heart failure (HF) will be defined by symptoms and/or signs of HF with evidence of diastolic or systolic dysfunction by echocardiography and elevated levels of natriuretic peptide (BNP >35 pg/mL and/or NT-proBNP >125 pg/mL).[3]
**Myocarditis, pericarditis, Takotsubo syndrome**	Myocarditis will be defined by chest pain, a rise in troponin or creatine kinase level above the 99 percentiles of the upper reference limit and confirmation by cardiovascular magnetic resonance (CMR) using the Lake Louis criteria.[4] Pericarditis will be defined when two out of the four following criteria are fulfilled: a) chest pain b) pericardial rubs c) ECG changes d) pericardial effusion. Takotsubo syndrome will be defined using the clinical expert consensus statement on takotsubo syndrome.[5]
**Pulmonary Embolism**	Symptoms of pulmonary embolism (dyspnea, chest pain…) confirmed by imaging tests.[6]
**Acute supraventricular**	Symptoms of tachycardia (palpitations, dyspnea…) leading to hospitalization in ICCU and 12-lead ECG confirming the

**arrhythmias**	supraventricular arrhythmia.
**Ventricular arrhythmias**	Symptoms of tachycardia (palpitations, dyspnea…) leading to hospitalization in ICCU and 12-lead ECG confirming sustained ventricular tachycardia.
**Other**	The subgroup "other diagnosis" includes all diagnoses not eligible for the above categories, including aortic dissection, coronary spasm, unstable angina, endocarditis, hypertensive emergency, acute chest pain without etiology.

Method 3. Absolute standardized mean differences in the propensity matched population (STEMI patients vs. NSTEMI patients)

Absolute standardized mean differences calculated using Yang and Dalton's method <0.2 were used as a proxy of covariate balance.

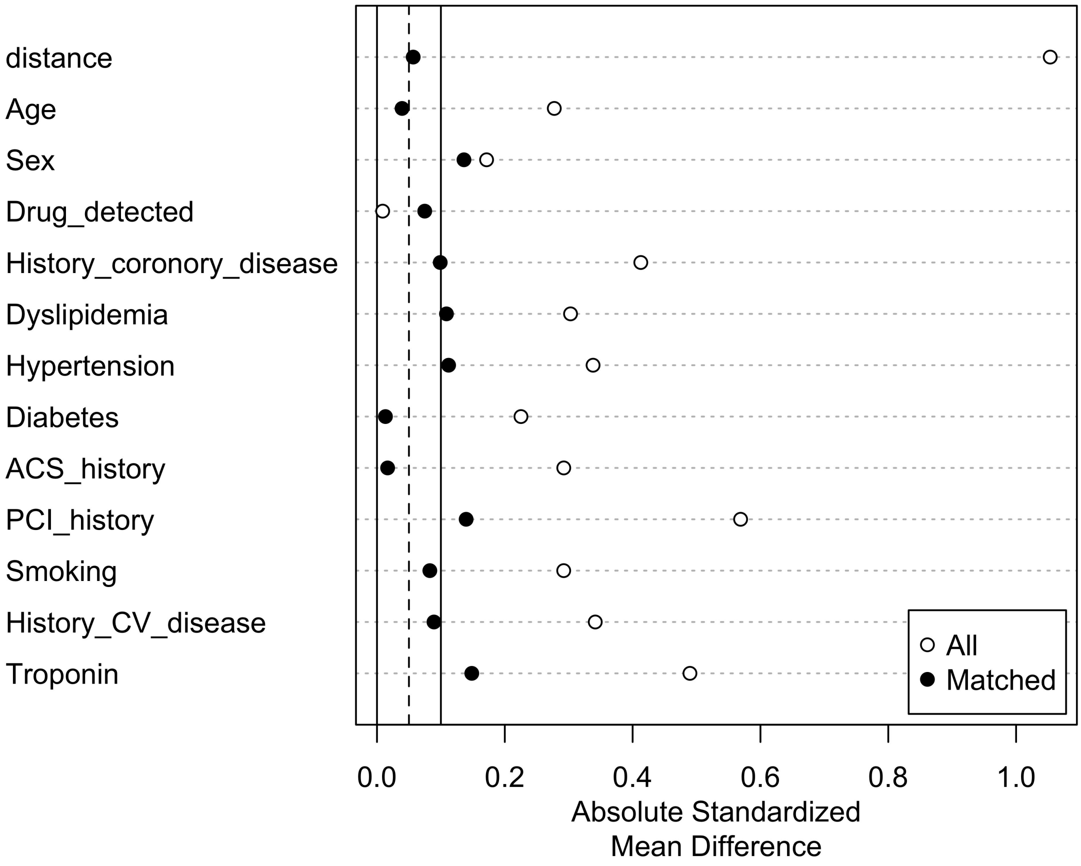

Method 4. Reasons for failure to perform drug urine assay

Among the 76 patients without drug urine assay performed, 38 (50%) patients had urine leakage in a diaper without the possibility of performing the drug urine assay efficiently, 28 (36.8%) patients did not urinate during the first 12 hours of hospitalization, and for 10 (13.2%) patients the drug urine assay was not performed by the paramedical team due to having prioritized the therapeutic management in emergency, or due to an oversight, especially at night.

Tables

Table 1. Detailed list of participating centers

This table reports all participating centers with the respective number of patients recruited.

Centers	Patients recruited (n)
Amiens, University Hospital Center	6
Annecy, Hospital Center	51
Avignon, Hospital Center	58
Bobigny, Avicenne University Hospital Center, AP-HP	9
Boulogne-Billancourt, Hôpital Ambroise Paré, University Hospital Center, AP-HP	10
Bordeaux, University Hospital Center	45
Brest, University Hospital Center	63
Caen, University Hospital Center	32
Chartres, Hospital Center	30
Créteil, Henri Mondor University Hospital Center	52
Dijon, University Hospital Center	69
Fréjus, Hospital Center	26
Grenoble, University Hospital Center	76
La Réunion, University Hospital Center	54
Lille, University Hospital Center	43
Limoges, University Hospital Center	6
Lyon, University Hospital Center	42
Marseille, La Timone University Hospital Center, AP-HM	51
Fort de France, University Hospital Center, Martinique	33
Montfermeil, Hospital Center	40
Montpellier, University Hospital Center	46
Montreuil, Hospital Center	32
Neuilly-sur-Seine, Ambroise Paré Private Hospital	42
Nîmes, University Hospital Center	56
Orléans, Regional Hospital Center	50
Paris, Hôpital Bichat, University Hospital Center, AP-HP	35
Paris, Hôpital Cochin, University Hospital Center, AP-HP	30

Paris, Hôpital Européen Georges Pompidou, University Hospital Center, AP-HP	12
Paris, Hôpital Lariboisière, University Hospital Center, AP-HP	60
Paris, Hôpital Saint-Antoine, University Hospital Center, AP-HP	27
Percy-Clamart, Hôpital d'Instruction des Armées	10
Poitiers, University Hospital Center	73
Rennes, University Hospital Center	42
Rouen, University Hospital Center	25
Saintes, Hospital Center	25
Strasbourg, University Hospital Center	84
Toulouse, University Hospital Center	83
Tours, Clinique Saint Gatien Alliance (NCT+), Saint-Cyr-sur-Loire	17
Versailles, Hôpital Mignot, Hospital Center	30
**Total**	1575

Abbreviations: AP-HM: Assistance Publique-Hôpitaux de Marseille; AP-HP: Assistance Publique-Hôpitaux de Paris; NCT+: La Nouvelle Clinique de Tours+.

Table 2. Reliability parameters of the urine drug assay

A) Technical methodology to perform the *NarcoCheck® urine drug assay*

The following recreational drugs were evaluated for all consecutive patients by urine drug assay using a cartridge-based system (NarcoCheck®, Kappa City Biotech SAS, Montluçon, France) as soon as possible at most within two hours of admission to the ICCU: i) cannabinoids (tetrahydrocannabinol [THC]), including cannabis and hashish; ii) cocaine and metabolites, including crack; iii) amphetamines; iv) MDMA; and v) heroin and other opioids. The test was performed using a urine jar or a urinary catheter by nurses who were trained following a standardized protocol just before the recruitment period to ensure maintenance of clinical accuracy of the procedure. The test is immersed directly into the urine sample, which prevents any liquid handling. Of note, morphine and other opioid administration for pain sedation during the initial management of patients before admission to the ICCU was recorded, and their urine tests for opioids were considered negative.

B) Reliability of the NarcoCheck® urine drug assay

To assess the reliability of the NarcoCheck® urine drug assay, a comparative analysis between NarcoCheck® and the findings of the regional reference Laboratory in Biological Toxicology was performed on a random sample of 60 patients. NarcoCheck® urine drug assay had a sensitivity of 91.7% and specificity of 97.9%.

		**Reference Laboratory in Biological Toxicology**	
		Positive	Negative
**NarcoCheck® urine drug assay**	Positive	11	1
	Negative	1	47

C) Technical methodology to perform the reference test in the Laboratory

The reference test for the presence of drugs in the regional reference Laboratory in Biological Toxicology is based on radioimmunoassay or enzyme immunoassay depending on the substance. Of note, the comparative analysis was performed for all patients with a positive NarcoCheck® urine drug assay with the indication to the reference laboratory to assess the presence of all the recreational drugs evaluated in the study regardless of the initial result using NarcoCheck®.

D) Detection limit of the method for each drug

The detection limit of the method for each drug is depicted just below (website of Kappa City Biotech SAS: https://www.narcocheck.com/en/multi-drugs-urine-tests/multi-drugs-medicinesurine-test-10in1.html):

Technical data

Cut-off : each substance is screened at a specific cut-off.

If the urine sample concentrates drug levels above the indicated cut-offs, the test will be positive for that drug, otherwise the test will be negative.

THC	50 ng/ml	BAR	300 ng/ml
COC	300 ng/ml	BZD	300 ng/ml
MOR	300 ng/ml	TCA	1000 ng/ml
AMP	1000 ng/ml	EDDP	100 ng/ml
MDMA	500 ng/ml	BUP	10 ng/ml

Table 3. List of the ADDICT-ICCU investigators (in alphabetical order)

First name	Last name	Center
Victor	ABOYANS	Department of Cardiology, University Hospital of Limoges, 87000 Limoges, France
Emeric	ALBERT	Department of Cardiology, Hôpital Européen Georges Pompidou (HEGP), AP-HP, 75015 Paris, France
Franck	ALBERT	Service de Cardiologie, Center Hospitalier de Chartres, 28630 Le Coudray, France
Sean	ALVAIN	Service de Cardiologie, Center Hospitalier de Saintonge, 17100 Saintes, France
Nabil	AMRI	Service de Cardiologie Interventionnelle, CHU Timone, AP-HM, Aix Marseille Université, Marseille, France
Stéphane	ANDRIEU	Service de Cardiologie, Hôpital Henri Duffaut, 84902 Avignon, France
Sabir	ATTOU	Department of Cardiology, Caen University Hospital, 14000 Caen, France
Simon	AUVRAY	Department of Cardiology, Felix-Guyon University Hospital, 97400 Saint-Denis-de-La-Reunion, France
Sonia	AZZAKANI	Department of Cardiology, University Hospital of Poitiers, 86000 Poitiers, France
Ruben	AZENCOT	Service de Cardiologie, Hôpital Cochin, AP-HP, 75014 Paris, France
Marc	BEDOSSA	CHU Rennes, Service de Cardiologie et Maladies Vasculaires, 35000 Rennes , France
Franck	BOCCARA	Department of Cardiology, Saint-Antoine Hospital, AP-HP, Sorbonne University, 75012 Paris, France
Albert	BOCCARA	Department of Cardiology, Andre Gregoire Hospital, 93100 Montreuil, France
Thomas	BOCHATON	Intensive Cardiological Care Division, Louis Pradel Hospital, Hospices Civils de Lyon, 69500 Bron, France

Eric	BONNEFOY-CUDRAZ	Intensive Cardiological Care Division, Louis Pradel Hospital, Hospices Civils de Lyon, 69500 Bron, France
Guillaume	BONNET	Service de Cardiologie Interventionnelle, CHU Timone, AP-HM, Aix Marseille Université, INSERM, INRAE, C2VN, 13500 Marseille, France
Guillaume	BONNET	Unité Médico-Chirurgical de Valvulopathies et Cardiomyopathies, Hôpital Cardiologique Haut-Lévêque, Center Hospitalier Universitaire de Bordeaux, 33600 Pessac, France
Nabil	BOUALI	Service de Cardiologie, Center Hospitalier de Saintonge, 17100 Saintes, France
Océane	BOUCHOT	Service de Cardiologie, Center Hospitalier Annecy Genevois, 74370 Epagny Metz-Tessy, France
Claire	BOULETI	Department of Cardiology, University Hospital of Poitiers, 86000 Poitiers, France
Tanissia	BOUKERTOUTA	Department of Cardiology, Hôpital Avicenne, AP-HP, 93000 Bobigny, France
Jean Baptiste	BRETTE	Cardiology Department, Rangueil University Hospital, 31000 Toulouse, France
Marjorie	CANU	Service de Cardiologie, CHU Grenoble-Alpes, 38700 La Tronche, France
Aures	CHAIB	Department of Cardiology, Andre Gregoire Hospital, 93100 Montreuil, France
Clement	CHARBONNEL	Service de Cardiologie, Hôpital Mignot, 78000 Versailles, France
Anne Solene	CHAUSSADE	Département de Cardiologie, Clinique Ambroise Paré, 92200 Neuilly-sur-Seine, France
Alexandre	COPPENS	Department of Cardiology, Andre Gregoire Hospital, 93100 Montreuil, France
Yves	COTTIN	Department of Cardiology, University Hospital, 21000 Dijon, France
Arthur	DARMON	Department of Cardiology, Hôpital Bichat, AP-HP,

		Université de Paris, 75018 Paris, France
Elena	DE ANGELIS	Intensive Cardiological Care Division, Louis Pradel Hospital, Hospices Civils de Lyon, 69500 Bron, France
Clément	DELMAS	Intensive Cardiac Care Unit, Rangueil University Hospital, 31000 Toulouse, France
Laura	DELSARTE	Department of Cardiology, University Hospital of Brest, 29609 Brest, France
Antoine	DENEY	Cardiology Department, Rangueil University Hospital, 31000 Toulouse, France
Jean Claude	DIB	Département de Cardiologie, Clinique Ambroise Paré, 92200 Neuilly-sur-Seine, France
Jean-Guillaume	DILLINGER	Department of Cardiology, Hôpital Lariboisière, AP-HP, Université de Paris-Cité, INSERM U-942, 75010 Paris, France
Clemence	DOCQ	Department of Cardiology, University Hospital of Lille, 59000 Lille, France
Valentin	DUPASQUIER	Department of Cardiology, CHU Montpellier, 34000 Montpellier, France
Meyer	ELBAZ	Cardiology Department, Rangueil University Hospital, 31000 Toulouse, France
Antony	EL HADAD	Service de Cardiologie, Hôpital Montfermeil, 93370 Montfermeil, France
Amine	EL OUAHIDI	Department of Cardiology, University Hospital of Brest, 29609 Brest, France
Nacim	EZZOUHAIRI	Cardiology Intensive Care Unit and Interventional Cardiology, Hôpital Cardiologique du Haut-Lévêque, 33600 Pessac, France
Julien	FABRE	Department of Cardiology, University Hospital of Martinique, 97261 Fort-de-France, France
Damien	FARD	Intensive Cardiac Care Unit University, Hospital Henri Mondor, 94000 Créteil, France
Charles	FAUVEL	Department of Cardiology, Rouen University

		Hospital, 76000 Rouen, France
Édouard	GERBAUD	Cardiology Intensive Care Unit and Interventional Cardiology, Hôpital Cardiologique du Haut-Lévêque, 33600 Pessac; and Bordeaux Cardio-Thoracic Research Center, U1045, Bordeaux University, 33000 Bordeaux, France
Martine	GILARD	Department of Cardiology, University Hospital of Brest, 29609 Brest, France
Marc	GORALSKI	Service de Cardiologie, Center Hospitalier d'Orleans, 45100 Orléans, France
Nissim	GRINBERG	Service de Cardiologie, Hôpital Mignot, 78000 Versailles, France
Alain	GRENTZINGER	Service de Cardiologie, Center Hospitalier de Saintonge, 17100 Saintes, France
Marie	HAUGUEL-MOREAU	Service de Cardiologie, Hôpital Ambroise Paré, University Hospital Center, AP-HP, 92012 Boulogne-Billancourt, France
Patrick	HENRY	Department of Cardiology, Hôpital Lariboisière, AP-HP, Université de Paris-Cité, INSERM U-942, 75010 Paris, France
Fabien	HUET	Department of Cardiology, CHU Montpellier, 34000 Montpellier, France
Thomas	LANDEMAINE	Unité de Soins Intensifs Cardiologiques, CHU Amiens, 80000 Amiens, France
Benoit	LATTUCA	Department of Cardiology, Nîmes University Hospital, Montpellier University, 30029 Nîmes, France
Léo	LEMARCHAND	CHU Rennes, Service de Cardiologie et Maladies Vasculaires, 35000 Rennes, France
Thomas	LEVASSEUR	Service de Cardiologie, Center Hospitalier de Fréjus/Saint-Raphaël, 83600 Fréjus, France
Pascal	LIM	Intensive Cardiac Care Unit, University Hospital Henri Mondor, 94000 Créteil, France

Laura	MAITRE BALLESTEROS	Service de Cardiologie, CHU Grenoble-Alpes, 38700 La Tronche, France
Nicolas	MANSENCAL	Service de Cardiologie, Hôpital Ambroise Paré, University Hospital Center, AP-HP, 92012 Boulogne Billancourt, France
Benjamin	MARIE	Cardiology Intensive Care Unit and Interventional Cardiology, Hôpital Cardiologique du Haut-Lévêque, 33600 Pessac, France
David	MARTINEZ	Department of Cardiology, Nîmes University Hospital, Montpellier University, 30029 Nîmes, France
Benoit	MERAT	Service de Cardiologie et Médecine Aéronautique, Hôpital d'Instruction des Armées Percy, 92140 Clamart, France
Christophe	MEUNE	Department of Cardiology, Hôpital Avicenne, AP-HP, 93000 Bobigny, France
Damien	MILLISCHER	Service de Cardiologie, Hôpital Montfermeil, 93370 Montfermeil, France
Thomas	MOINE	NCT+, 37540 Saint-Cyr-sur-Loire, France
Pascal	NHAN	Department of Cardiology, Saint-Antoine Hospital, AP-HP, Sorbonne University, 75012 Paris, France
Nathalie	NOIRCLERC	Service de Cardiologie, Center Hospitalier Annecy Genevois, 74370 Epagny Metz-Tessy, France
Patrick	OHLMANN	Department of Cardiovascular Medicine, Nouvel Hôpital Civil, Strasbourg University Hospital, 67000 Strasbourg, France
Théo	PEZEL	Department of Cardiology, Hôpital Lariboisière, AP-HP, Université de Paris-Cité, INSERM U-942, 75010 Paris, France
Fabien	PICARD	Service de Cardiologie, Hôpital Cochin, AP-HP, 75014 Paris, France
Nicolas	PILIERO	Service de Cardiologie, CHU Grenoble-Alpes, 38700 La Tronche, France

Thibaut	POMMIER	Department of Cardiology, University Hospital, 21000 Dijon, France
Etienne	PUYMIRAT	Department of Cardiology, Hôpital Européen Georges Pompidou (HEGP), AP-HP, 75015 Paris, France
Arthur	RAMONATXO	Department of Cardiology, University Hospital of Poitiers, 86000 Poitiers, France
Reza	ROSSANALY VASRAM	Department of Cardiology, Felix-Guyon University Hospital, 97400 Saint-Denis-de-La-Reunion, France
François	ROUBILLE	Department of Cardiology, CHU Montpellier, 34000 Montpellier, France
Vincent	ROULE	Department of Cardiology, Caen University Hospital, 14000 Caen, France
Guillaume	SCHURTZ	Department of Cardiology, University Hospital of Lille, 59000 Lille, France
Mathilde	STEVENARD	Service de Cardiologie et Médecine Aéronautique, Hôpital d'Instruction des Armées Percy, 92140 Clamart, France
David	SULMAN	Department of Cardiology, Hôpital Bichat, AP-HP, Université de Paris, 75018 Paris, France
Fédérico	SWEDSKY	Service de Cardiologie, Hôpital Henri Duffaut, 84902 Avignon, France
Victoria	TEA	Department of Cardiology, Hôpital Européen Georges Pompidou (HEGP), AP-HP, 75015 Paris, France
Eugénie	THEVENET	Department of Cardiology, University Hospital of Martinique, 97261 Fort-de-France, France
Christophe	THUAIRE	Service de Cardiologie, Center Hospitalier de Chartres, 28630 Le Coudray, France
Antonin	TRIMAILLE	Department of Cardiovascular Medicine, Nouvel Hôpital Civil, Strasbourg University Hospital, 67000 Strasbourg, France
Christophe	TRON	Department of Cardiology, Rouen University Hospital, 76000 Rouen, France
Guillaume	VIBOUD	Unité de Soins Intensifs Cardiologiques, CHU

		Amiens, 80000 Amiens, France
Dominique	YOMI	Service de Cardiologie, Center Hospitalier de Fréjus/Saint-Raphaël, 83600 Fréjus, France
Cyril	ZAKINE	Clinique Saint Gatien Alliance (NCT+), 37540 Saint-Cyr-sur-Loire, France

AP-HM: Assistance Publique-Hôpitaux de Marseille; AP-HP: Assistance Publique-Hôpitaux de Paris; NCT+: La Nouvelle Clinique de Tours+.

Table 4. Baseline characteristics of the overall ACS population according to recreational drug detection.

	**Overall ACS population (N=712)**	**No recreational drug detected (N=616)**	**Recreational drug detected (N=96)**	**p-value**
Age, years	64±13	65 ± 12	54 ± 13	**<0.001**
Men, n (%)	531 (74.6)	449 (72.9)	82 (85.4)	**0.009**
Body mass index, kg/m²	27.3 ± 5.2	27.4 ± 5.2	26.2 ± 5.1	**0.012**
Admission diagnosis				0.907
NSTEMI	404 (56.7)	349 (56.7)	55 (57.3)	
STEMI	308 (43.3)	267 (43.3)	41 (42.7)	
**CV risk factors**, n (%)				
Hypertension	394 (55.3)	361 (58.6)	33 (34.4)	**<0.001**
Diabetes	162 (22.8)	152 (24.7)	10 (10.4)	**0.002**
Dyslipidemia	286 (40.2)	252 (40.9)	34 (35.4)	0.307
Family history of CAD	151 (21.2)	127 (20.6)	24 (25.0)	0.328
Smoking	231 (32.4)	168 (27.3)	63 (65.6)	**<0.001**
**Medical history of CV disease**, n (%)				

History of ACS	110 (15.4)	95 (15.4)	15 (15.6)	0.959
Previous PCI	429 (60.3)	368 (59.7)	61 (63.5)	0.479
History of coronary disease[a]	491 (69.0)	424 (68.8)	67 (69.8)	0.850
History of CKD[b]	51 (7.2)	47 (7.6)	4 (4.2)	0.221
History of CV disease[c]	516 (72.5)	448 (72.7)	68 (70.8)	0.699
Alcohol consumption	403 (57.6)	345 (57.0)	58 (61.1)	0.460
**Clinical data on admission**				
Systolic blood pressure, mm Hg	136 ± 26	137 ± 26	131 ± 24	**0.025**
Heart rate, beats/min	79 ± 18	78 ± 17	85 ± 21	**<0.001**
Oxygen saturation, %	97.3 ± 4.4	97.4 ± 2.5	96.7 ± 10.2	0.194
ICCU hospitalization duration, days	6.9 ± 15.8	7.0 ± 16.9	6.2 ± 4.8	0.264
Killip class				
1	649 (91.3)	561 (91.2)	88 (91.7)	
≥ 2	62 (8.7)	54 (8.8)	8 (8.3)	0.885
CHF signs	123 (17.3)	104 (16.9)	19 (19.8)	0.483
**Laboratory results**				
Hemoglobin, g/dL	13.9 ± 1.8	13.8 ± 1.8	14.4 ± 1.7	**0.002**
Creatininemia, mmol/L	91 ± 64	92 ± 68	82 ± 28	0.917
High-sensitivity cardiac troponin peak[d]	638 ± 1523	634 ± 1575	662 ± 1130	0.145
BNP, pg/mL	289 ± 613	308 ± 645	147 ± 245	0.093
**Echocardiography data**				

LV ejection fraction, %	53 ± 11	53 ± 11	52 ± 11	0.273
TAPSE, mm	21.5 ± 4.1	21.6 ± 4.0	20.7 ± 4.8	**0.040**
sPAP, mmHg	32 ± 12	30 ± 11	39 ± 13	**<0.001**
TAPSE/sPAP ratio	0.8 ± 0.4	0.8 ± 0.4	0.6 ± 0.2	**<0.001**
VTI, cm	19.4 ± 4.8	19.6 ± 4.7	18.1 ± 4.8	**0.002**
E/e' ratio	8.3 ± 3.0	8.4 ± 2.9	8.2 ± 3.5	0.255
E/A ratio	1.0 ± 0.5	1.0 ± 0.5	1.2 ± 0.5	**0.001**

Values are n (%), mean ± SD, or median (interquartile range).

[a]History of coronary disease defined by the presence of ³1 of the following: ACS, angioplasty, or coronary bypass.

[b]CKD defined by history of chronic kidney disease with glomerular filtration rate <60 mL/min/m^2.

[c]CV disease defined by the presence of known MI, previous PCI, previous CABG, peripheral atheroma with revascularization, stroke, history of heart failure, history of atrial fibrillation, history of surgery for valvular heart disease, pacemaker or ICD, and cardiomyopathies.

[d]Represents the n-fold troponin levels.

Abbreviations: ACS: acute coronary syndrome; BNP: B-type natriuretic peptide; CABG: coronary artery bypass graft; CAD: coronary artery disease; CHF: congestive heart failure; CKD: chronic kidney disease; CV: cardiovascular; ICCU: intensive cardiac care unit; ICD: Implantable Cardioverter-Defibrillator; LV: left ventricle; MI: myocardial infarction; NSTEMI: non-ST-segment elevation myocardial infarction; PCI: percutaneous coronary intervention; SD: standard deviation; sPAP: systolic pulmonary artery pressure; STEMI: ST-segment elevation myocardial infarction; TAPSE: tricuspid annular plane systolic excursion; VTI: velocity time integral.

Table 5. Baseline characteristics of the overall ACS population according to MACE.

	**Overall ACS population (N=712)**	**No MACE (N=662)**	**MACE (N=50)**	**p-value**
Age, years	64 ± 13	64 ± 12	66 ± 16	0.058
Men, n (%)	531 (74.6)	492 (74.3)	39 (78.0)	0.564
Body mass index, kg/m²	27.3 ± 5.2	27.3 ± 5.1	26.2 ± 5.8	0.075
Admission diagnosis				**0.029**
NSTEMI	404 (56.7)	383 (57.9)	21 (42.0)	
STEMI	308 (43.3)	279 (42.1)	29 (58.0)	
**CV risk factors**, n (%)				
Hypertension	394 (55.3)	368 (55.6)	26 (52.0)	0.623
Diabetes	162 (22.8)	149 (22.5)	13 (26.0)	0.570
Dyslipidemia	286 (40.2)	263 (39.7)	23 (46.0)	0.383
Family history of CAD	151 (21.2)	140 (21.1)	11 (22.0)	0.887
Smoking	231 (32.4)	214 (32.3)	17 (34.0)	0.807
**Medical history of CV disease**, n (%)				

History of ACS	110 (15.4)	102 (15.4)	8 (16.0)	0.911
Previous PCI	429 (60.3)	399 (60.3)	30 (60.0)	0.970
History of coronary disease[a]	491 (69.0)	456 (68.9)	35 (70.0)	0.869
History of CKD[b]	51 (7.2)	46 (6.9)	5 (10.0)	0.393
History of CV disease[c]	516 (72.5)	480 (72.5)	36 (72.0)	0.938
Alcohol consumption	403 (57.6)	380 (58.4)	23 (46.9)	0.118
**Clinical data on admission**				
Systolic blood pressure, mm Hg	136 ± 26	137 ± 26	134 ± 25	0.358
Heart rate, beats/min	79 ± 18	79 ± 18	80 ± 15	0.486
Oxygen saturation, %	97.3 ± 4.4	97.3 ± 4.6	97.4 ± 2.1	0.815
ICCU hospitalization duration, days	6.9 ± 15.8	6.7 ± 14.6	9.7 ± 27.9	0.208
Killip class				**0.032**
1	649 (91.3)	608 (92.0)	41 (82.0)	
≥ 2	62 (8.7)	53 (8.0)	9 (18.0)	
CHF signs	123 (17.3)	108 (16.3)	15 (30.0)	**0.014**
**Urine drug assay**				
Drug detection	96 (13.5)	84 (12.7)	12 (24.0)	**0.024**
Cannabis	86 (12.1)	76 (11.5)	10 (20.0)	0.075
Stimulants[d]	18 (2.5)	16 (2.4)	2 (4.0)	0.365
Opioids[e]	34 (4.8)	25 (3.8)	9 (18.0)	**<0.001**
Depressants[f]	8 (1.1)	7 (1.1)	1 (2.0)	0.443

**Laboratory results**				
Hemoglobin, g/dL	13.9 ± 1.8	13.9 ± 1.7	13.8 ± 2.1	0.380
Creatininemia, mmol/L	91 ± 64	89 ± 59	113 ± 115	**0.029**
High-sensitivity cardiac troponin peak[g]	638 ± 1523	641 ± 1556	599 ± 983	0.374
BNP, pg/mL	289 ± 613	275 ± 564	475 ± 1092	0.051
**Echocardiography data**				
LV ejection fraction, %	53 ± 11	53 ± 11	50 ± 9	**0.017**
TAPSE, mm	21.5 ± 4.1	21.6 ± 4.1	20.1 ± 4.0	**0.024**
sPAP, mm Hg	32 ± 12	31 ± 12	37 ± 12	**0.002**
TAPSE/sPAP ratio	0.8 ± 0.4	0.8 ± 0.4	0.6 ± 0.2	**<0.001**
VTI, cm	19.4 ± 4.8	19.5 ± 4.7	18.7 ± 5.2	0.268
E/e ratio	8.3 ± 3.0	8.3 ± 3.0	9.1 ± 3.7	0.284
E/A ratio	1.0 ± 0.5	1.0 ± 0.5	1.1 ± 0.4	0.166

Values are n (%), mean ± SD, or median (interquartile range).

[a]*History of coronary disease defined by the presence of ³1 of the following: ACS, angioplasty, or coronary bypass.*

[b]*CKD defined by history of chronic kidney disease with glomerular filtration rate <60 mL/min/m^2.*

[c]*CV disease defined by the presence of known MI, previous PCI, previous CABG, peripheral atheroma with revascularization, stroke, history of heart failure, history of atrial fibrillation, history of surgery for valvular heart disease, pacemaker or ICD, and cardiomyopathies.*

[d]*Stimulants tested for: cocaine, amphetamine, and MDMA.*

[e]*Opioids tested for: morphine, buprenorphine, and EDDP.*

[f]*Depressants tested for: benzodiazepine and barbiturate.*

[g]*Represents the n-fold troponin levels.*

Abbreviations: ACS: acute coronary syndrome; BNP: B-type natriuretic peptide; CABG: coronary artery bypass graft; CAD: coronary artery disease; CHF: congestive heart failure; CKD: chronic kidney disease; CV: cardiovascular; EDDP: 2-Ethylidene-1,5-Dimethyl-3,3-Diphenylpyrrolidine; ICCU: intensive cardiac care unit; ICD: Implantable Cardioverter-Defibrillator; LV: left ventricle; MACE: major adverse cardiovascular event; MDMA: 3,4-Methylenedioxymethamphetamine; MI: myocardial infarction NSTEMI: non-ST-segment elevation myocardial infarction; PCI: percutaneous coronary intervention; SD: standard deviation; sPAP: systolic pulmonary artery pressure; STEMI: ST-segment elevation myocardial infarction; TAPSE: tricuspid annular plane systolic excursion; VTI: velocity time integral.

Table 6. Univariable and multivariable analyses of recreational drug use for MACE in the ACS population (N=712) according to traditional prognostic factors.

	**Univariable**		**Multivariable**[a]	
	**HR (95% CI)**	**p-value**	**HR (95% CI)**	**p-value**
Age, years	1.02 (1.00-1.04)	0.118	1.03 (1.01-1.06)	**0.009**
Men, n (%)	1.22 (0.62-2.38)	0.563	1.25 (0.63-2.47)	0.52
Body mass index, kg/m²	0.95 (0.90-1.02)	0.142	-	-
Admission diagnosis				
NSTEMI	-	-	-	-
STEMI	1.85 (1.05-3.24)	**0.032**	2.12 (1.19-3.77)	**0.010**
**CV risk factors**, n (%)				
Hypertension	0.87 (1.07-1.66)	0.635	-	-
Diabetes	1.21 (0.64-2.27)	0.559	1.36 (0.71-2.60)	0.37
Dyslipidemia	1.29 (0.74-2.25)	0.372	-	-
Family history of CAD	1.04 (0.53-2.04)	0.900	-	-
Smoking	1.07 (0.60-1.92)	0.819	1.12 (0.56-2.25)	0.76
**Medical history of CV disease**, n (%)			-	-
History of ACS	1.05 (0.49-2.24)	0.894	-	-
Previous PCI	0.99 (0.56-1.75)	0.984	-	-

History of coronary disease[b]	1.07 (0.58-1.95)	0.833	-	-
History of CKD[c]	1.48 (0.59-3.72)	0.408	-	-
History of CV disease[d]	0.99 (0.53-1.83)	0.969	-	-
Alcohol consumption	0.64 (0.37-1.13)	0.124	-	-
**Clinical data on admission**				
Systolic blood pressure, mm Hg	1.00 (0.99-1.01)	0.536	-	-
Heart rate, beats/min	1.00 (0.99-1.02)	0.654	-	-
Oxygen saturation, %	1.01 (0.93-1.10)	0.821	-	-
ICCU hospitalization duration, days	1.01 (1.00-1.02)	0.200	-	-
Killip class		**0.018**		
1	-		-	-
≥ 2	2.38 (1.16-4.90)		-	-
CHF signs	2.09 (1.14-3.83)	**0.017**	-	-
**Urine drug assay**				
Drug detection	2.08 (1.09-3.98)	**0.027**	3.01 (1.45-6.21)	**0.006**
Cannabis	1.87 (0.93-3.73)	0.077	-	-
Stimulants[e]	1.60 (0.39-6.57)	0.517	-	-
Opioids[f]	4.81 (2.34-9.91)	**<0.001**	-	-
Depressants[g]	1.76 (0.24-12.7)	0.576	-	-
**Laboratory results**				
Hemoglobin, g/dL	0.95 (0.81-1.12)	0.558	-	-

Creatininemia, mmol/L	1.00 (1.00-1.01)	**0.014**	-	-
High-sensitivity cardiac troponin peak[h]	1.00 (1.00-1.00)	0.882	-	-
BNP, pg/mL	1.00 (1.00-1.00)	0.175		
**Echocardiography data**				
LV ejection fraction, %	0.98 (0.95-1.00)	**0.047**	-	-
TAPSE, mm	0.92 (0.86-0.99)	**0.023**	-	-
sPAP, mm Hg	1.03 (1.01-1.05)	**0.005**	-	-
TAPSE/sPAP ratio	0.10 (0.03-0.39)	**<0.001**	-	-
VTI, cm	0.97 (0.90-1.03)	0.314	-	-
E/e ratio	1.08 (0.99-1.19)	0.097	-	-
E/A ratio	1.26 (0.70-2.29)	0.446	-	-

Bold values indicate the 2-tailed P-value reached statistical significance (<0.05).

[a]*Covariates determined by traditional prognostic factors: age, sex, Admission diagnosis, drug detected, diabetes, and smoking.*

[b]*History of coronary disease defined by the presence of ³1 of the following: ACS, angioplasty, or coronary bypass.*

[c]*CKD defined by history of chronic kidney disease with glomerular filtration rate <60 mL/min/m²*.

[d]*CV disease defined by the presence of known MI, previous PCI, previous CABG, peripheral atheroma with revascularization, stroke, history of heart failure, history of atrial fibrillation, history of surgery for valvular heart disease, pacemaker or ICD, and cardiomyopathies.*

[e]*Stimulants tested for: cocaine, amphetamine, and MDMA.*

[f]*Opioids tested for: morphine, buprenorphine, and EDDP.*

[g]*Depressants tested for: benzodiazepine and barbiturate.*

[h]*Represents the n-fold troponin levels.*

<u>Abbreviations</u>: ACS: acute coronary syndrome; BNP: B-type natriuretic peptide; CABG: coronary artery bypass graft; CAD: coronary artery disease; CHF: congestive heart failure; CI: confidence interval; CKD: chronic kidney disease; CV: cardiovascular; EDDP: 2-Ethylidene-1,5-Dimethyl-3,3-Diphenylpyrrolidine; HR: hazard ratio; ICCU: intensive cardiac care unit; ICD: Implantable Cardioverter-Defibrillator; Inf: infinity; LV: left ventricle; MACE: major adverse cardiovascular events; MDMA: 3,4-Methylenedioxymethamphetamine; MI: myocardial infarction; NSTEMI: non-ST-segment elevation myocardial infarction; PCI: percutaneous coronary intervention; sPAP: systolic pulmonary artery pressure; STEMI: ST-segment elevation myocardial infarction; TAPSE: tricuspid annular plane systolic excursion; VTI: velocity time integral.

Table 7. Baseline characteristics of the ACS population and the NSTEMI and STEMI subpopulations according to MACE.

	**Overall ACS population (N=712)**	**NSTEMI population (N=404)**			**STEMI population (N=308)**		
		**No MACE (N=383)**	**MACE (N=21)**	**p-value**	**No MACE (N=279)**	**MACE (N=29)**	**p-value**
Age, years	64 ± 13	65 ± 12	71 ± 13	**0.028**	62 ± 13	63 ± 17	0.319
Men, n (%)	531 (74.6)	275 (71.8)	14 (66.7)	0.612	217 (77.8)	25 (86.2)	0.292
Body mass index, kg/m²	27.3 ± 5.2	27.4 ± 5.4	28.1 ± 5.4	0.284	27.3 ± 4.7	24.8 ± 5.8	**0.001**
**CV risk factors**, n (%)							
Hypertension	394 (55.3)	237 (61.9)	16 (76.2)	0.187	131 (47.0)	10 (34.5)	0.200
Diabetes	162 (22.8)	98 (25.6)	9 (42.9)	0.081	51 (18.3)	4 (13.8)	0.548
Dyslipidemia	286 (40.2)	172 (44.9)	15 (71.4)	**0.018**	91 (32.6)	8 (27.6)	0.581
Family history of CAD	151 (21.2)	78 (20.4)	5 (23.8)	0.781	62 (22.2)	6 (20.7)	0.850
Smoking	231 (32.4)	103 (26.9)	3 (14.3)	0.201	111 (39.8)	14 (48.3)	0.375
**Medical history of CV disease**, n (%)							
History of ACS	110 (15.4)	72 (18.8)	6 (28.6)	0.262	30 (10.8)	2 (6.9)	0.752
Previous PCI	429 (60.3)	192 (50.1)	8 (38.1)	0.283	207 (74.2)	22 (75.9)	0.845
History of coronary disease[a]	491 (69.0)	236 (61.6)	13 (61.9)	0.979	220 (78.9)	22 (75.9)	0.709
History of CKD[b]	51 (7.2)	36 (9.4)	4 (19.0)	0.143	10 (3.6)	1 (3.4)	>0.999

History of CV disease[c]	516 (72.5)	256 (66.8)	13 (61.9)	0.641	224 (80.3)	23 (79.3)	0.900
Alcohol consumption	403 (57.6)	211 (55.8)	9 (42.9)	0.245	169 (61.9)	14 (50.0)	0.219
**Clinical data on admission**							
Systolic blood pressure, mm Hg	136 ± 26	143 ± 25	148 ± 27	0.452	128 ± 23	124 ± 17	0.466
Heart rate, beats/min	79 ± 18	78 ± 18	77 ± 14	0.908	80 ± 19	82 ± 16	0.592
Oxygen saturation, %	97.3 ± 4.4	97.1 ± 5.6	97.1 ± 2.1	0.486	97.5 ± 2.5	97.6 ± 2.1	0.871
ICCU hospitalization duration, days	6.9 ± 15.8	6.8 ± 15.4	6.0 ± 4.3	0.698	6.5 ± 13.4	12.4 ± 36.7	0.418
Killip class				**0.024**			0.319
1	649 (91.3)	354 (92.4)	16 (76.2)		254 (91.4)	25 (86.2)	
≥ 2	62 (8.7)	29 (7.6)	5 (23.8)		24 (8.6)	4 (13.8)	
CHF signs	123 (17.3)	65 (17.0)	6 (28.6)	0.232	43 (15.4)	9 (31.0)	0.063
**Urine drug assay**							
Drug detection	96 (13.5)	52 (13.6)	3 (14.3)	>0.999	32 (11.5)	9 (31.0)	**0.007**
Cannabis	86 (12.1)	47 (12.3)	2 (9.5)	>0.999	29 (10.4)	8 (27.6)	**0.013**
Stimulants[d]	18 (2.5)	12 (3.1)	1 (4.8)	0.506	4 (1.4)	1 (3.4)	0.392
Opioids[e]	34 (4.8)	11 (2.9)	2 (9.5)	0.142	14 (5.0)	7 (24.1)	**0.001**
Depressants[f]	8 (1.1)	2 (0.5)	0 (0.0)	>0.999	5 (1.8)	1 (3.4)	0.450
**Laboratory results**							
Hemoglobin, g/dL	13.9 ± 1.8	13.8 ± 1.8	13.4 ± 2.4	0.255	14.1 ± 1.7	14.1 ± 1.8	0.741

Creatininemia, mmol/L	91 ± 64	94 ± 67	116 ± 113	0.308	83 ± 44	111 ± 118	**0.024**
High-sensitivity cardiac troponin peak[g]	638 ± 1523	163 ± 445	87 ± 119	0.768	1303 ± 2181	1026 ± 1174	0.913
BNP, pg/mL	289 ± 613	340 ± 630	687 ± 1384	0.071	195 ± 459	158 ± 188	0.523
**Echocardiography data**							
LV ejection fraction, %	53 ± 11	55 ± 11	52 ± 8	**0.041**	50 ± 11	48 ± 10	0.425
TAPSE, mm	21.5 ± 4.1	21.7 ± 4.3	21.2 ± 4.0	0.550	21.4 ± 3.9	19.3 ± 3.8	**0.019**
sPAP, mm Hg	32 ± 12	32 ± 12	37 ± 16	0.214	30 ± 11	36 ± 8	**0.001**
TAPSE/sPAP ratio	0.8 ± 0.4	0.8 ± 0.4	0.6 ± 0.2	0.163	0.8 ± 0.4	0.6 ± 0.2	**<0.001**
VTI, cm	19.4 ± 4.8	20.0 ± 5.1	20.2 ± 6.3	0.663	18.7 ± 4.0	17.8 ± 4.2	0.507
E/e ratio	8.3 ± 3.0	8.3 ± 3.0	9.8 ± 2.7	**0.042**	8.2 ± 2.9	8.7 ± 4.2	0.875
E/A ratio	1.0 ± 0.5	1.0 ± 0.5	1.2 ± 0.4	0.121	1.0 ± 0.4	1.0 ± 0.4	0.618

Values are n (%), mean ± SD, or median (interquartile range).

[a]*History of coronary disease defined by the presence of ³1 of the following: ACS, angioplasty, or coronary bypass.*

[b]*CKD defined by history of chronic kidney disease with glomerular filtration rate <60 mL/min/m*^{2}*.*

[c]*CV disease defined by the presence of known MI, previous PCI, previous CABG, peripheral atheroma with revascularization, stroke, history of heart failure, history of atrial fibrillation, history of surgery for valvular heart disease, pacemaker or ICD, and cardiomyopathies.*

[d]*Stimulants tested for: cocaine, amphetamine, and MDMA.*

[e]*Opioids tested for: morphine, buprenorphine, and EDDP.*

[f]*Depressants tested for: benzodiazepine and barbiturate.*

[g]*Represents the n-fold troponin levels.*

Abbreviations: ACS: acute coronary syndrome; BNP: B-type natriuretic peptide; CABG: coronary artery bypass graft; CAD: coronary artery disease; CHF: congestive heart failure; CKD: chronic kidney disease; CV: cardiovascular; EDDP: 2-Ethylidene-1,5-Dimethyl-3,3-Diphenylpyrrolidine; ICCU: intensive cardiac care unit; ICD: Implantable Cardioverter-Defibrillator; LV: left ventricle; MACE: major adverse cardiovascular event; MDMA: 3,4-Methylenedioxymethamphetamine; MI: myocardial infarction NSTEMI: non-ST-segment elevation myocardial infarction; PCI: percutaneous coronary intervention; SD: standard deviation; sPAP: systolic pulmonary artery pressure; STEMI: ST-segment elevation myocardial infarction; TAPSE: tricuspid annular plane systolic excursion; VTI: velocity time integral.

Table 8. Univariable analyses of recreational drug detected for MACE in NSTEMI and STEMI patients before propensity score matching.

	**NSTEMI (N=404)**		**STEMI (N=308)**	
	**Hazard Ratio (95% CI)**	**p-value**	**Hazard Ratio (95% CI)**	**p-value**
Age, years	1.05 (1.01-1.09)	**0.022**	1.01 (0.98-1.04)	0.570
Men, n (%)	0.79 (0.32-1.97)	0.619	1.74 (0.61-5.01)	0.302
Body mass index, kg/m²	1.02 (0.95-1.10)	0.543	0.88 (0.81-0.97)	**0.007**
**CV risk factors**, n (%)				
Hypertension	1.95 (0.71-5.32)	0.193	0.61 (0.28-1.31)	0.206
Diabetes	2.18 (0.92-5.16)	0.078	0.71 (0.25-2.04)	0.522
Dyslipidemia	3.00 (1.16-7.73)	**0.023**	0.80 (0.35-1.80)	0.585
Family history of CAD	1.20 (0.44-3.26)	0.728	0.92 (0.38-2.27)	0.862
Smoking	0.46 (0.14-1.56)	0.214	1.38 (0.67-2.86)	0.388
**Medical history of CV disease**, n (%)				
History of ACS	1.71 (0.66-4.41)	0.267	0.63 (0.15-2.66)	0.531
Previous PCI	0.62 (0.26-1.49)	0.285	1.11 (0.48-2.60)	0.806
History of coronary disease[a]	1.02 (0.42-2.46)	0.964	0.87 (0.37-2.04)	0.751
History of CKD[b]	2.26 (0.76-6.70)	0.143	0.93 (0.13-6.80)	0.940

History of CV disease[c]	0.82 (0.34-1.91)	0.632	0.03 (0.00-0.24)	**<0.001**
Alcohol consumption	0.60 (0.25-1.42)	0.245	0.64 (0.31-1.35)	0.240
**Clinical data on admission**				
Systolic blood pressure, mm Hg	1.01 (0.99-1.02)	0.359	0.99 (0.98-1.01)	0.428
Heart rate, beats/min	1.00 (0.97-1.02)	0.887	1.01 (0.99-1.02)	0.593
Oxygen saturation, %	1.00 (0.92-1.08)	0.982	1.02 (0.87-1.20)	0.784
ICCU hospitalization duration, days	1.00 (0.95-1.04)	0.822	1.01 (1.00-1.02)	0.076
Killip class				
1	-	**-**	-	**-**
≥ 2	3.58 (1.31-9.79)	**0.013**	1.61 (0.56-4.64)	0.375
CHF signs	1.92 (0.74-4.94)	0.178	2.26 (1.03-4.96)	**0.043**
**Urine drug assay**				
Drug detection	1.06 (0.31-3.59)	0.929	3.13 (1.42-6.87)	**0.004**
Cannabis	0.76 (0.18-3.26)	0.712	2.99 (1.32-16.76)	**0.008**
Stimulants[d]	1.50 (0.20-11.2)	0.692	2.17 (0.30-16.0)	0.447
Opioids[e]	3.37 (0.78-14.5)	0.102	4.88 (2.08-11.4)	**<0.001**
Depressants[f]	Inf	0.998	1.75 (0.24-12.9)	0.581
**Laboratory results**				
Hemoglobin, g/dL	0.88 (0.70-1.11)	0.269	1.00 (0.80-1.24)	0.976
Creatininemia, mmol/L	1.00 (1.00-1.01)	0.140	1.00 (1.00-1.01)	0.350
High-sensitivity cardiac troponin peak[g]	1.00 (1.00-1.00)	0.461	1.00 (1.00-1.00)	0.565

BNP, pg/mL	1.00 (1.00-1.00)	0.116	1.00 (1.00-1.00)	0.820
**Echocardiography data**				
LV ejection fraction, %	0.98 (0.94-1.01)	0.179	0.98 (0.95-1.02)	0.350
TAPSE, mm	0.98 (0.88-1.09)	0.672	0.86 (0.77-0.96)	**0.008**
sPAP, mm Hg	1.03 (1.00-1.06)	0.092	1.04 (1.01-1.07)	**0.017**
TAPSE/sPAP ratio	0.25 (0.04-1.45)	0.123	0.03 (0.00-0.24)	**<0.001**
VTI, cm	1.01 (0.92-1.10)	0.903	0.95 (0.86-1.04)	0.266
E/e ratio	1.15 (0.98-1.34)	0.078	1.05 (0.93-1.18)	0.419
E/A ratio	1.49 (0.66-3.38)	0.337	1.12 (0.46-2.68)	0.807

Bold values indicate the 2-tailed P-value reached statistical significance (<0.05).

[a]*History of coronary disease defined by the presence of* ^{3}*1 of the following: ACS, angioplasty, or coronary bypass.*

[b]*CKD defined by history of chronic kidney disease with glomerular filtration rate <60 mL/min/*m^2*.*

[c]*CV disease defined by the presence of known MI, previous PCI, previous CABG, peripheral atheroma with revascularization, stroke, history of heart failure, history of atrial fibrillation, history of surgery for valvular heart disease, pacemaker or ICD, and cardiomyopathies.*

[d]*Stimulants tested for: cocaine, amphetamine, and MDMA.*

[e]*Opioids tested for: morphine, buprenorphine, and EDDP.*

[f]*Depressants tested for: benzodiazepine and barbiturate.*

[g]*Represents the n-fold troponin levels.*

Abbreviations: ACS: acute coronary syndrome; BNP: B-type natriuretic peptide; CABG: coronary artery bypass graft, CAD: coronary artery disease; CHF: congestive heart failure; CI: confidence interval; CKD: chronic kidney disease; CV: cardiovascular; EDDP: 2-Ethylidene-1,5-

Dimethyl-3,3-Diphenylpyrrolidine; HR: hazard ratio; ICCU: intensive cardiac care unit; ICD: Implantable Cardioverter-Defibrillator; Inf; infinity; LV: left ventricle; MDMA: 3,4-Methylenedioxymethamphetamine; MI: myocardial infarction; NSTEMI: non-ST-segment elevation myocardial infarction; PCI: percutaneous coronary intervention; sPAP: systolic pulmonary artery pressure; STEMI: ST-segment elevation myocardial infarction; TAPSE: tricuspid annular plane systolic excursion; VTI: velocity time integral.

Table 9. Multivariable analyses of recreational drug detected for MACE in NSTEMI and STEMI patients according to traditional prognostic factors.

	NSTEMI[a] (N=404)		STEMI[a] (N=308)	
	**HR (95% CI)**	**p-value**	**HR (95% CI)**	**p-value**
Age	1.04 (1.00-1.09)	**0.045**	1.03 (1.00-1.07)	0.054
Men, n (%)	1.00 (0.39-2.54)	>0.99	1.65 (0.56-4.83)	0.34
Drug detected	1.61 (0.45-5.71)	0.48	4.05 (1.60-10.3)	**0.005**
Smoking	0.69 (0.18-2.69)	0.59	1.28 (0.54-3.04)	0.58

Bold values indicate the 2-tailed P-value reached statistical significance (<0.05).

[a]Covariates determined by traditional prognostic factors: age, sex, drug detected, smoking.

Abbreviations: CI: confidence interval; HR: hazard ratio; MACE: major adverse cardiovascular events; NSTEMI: non-ST-segment elevation myocardial infarction; STEMI: ST-segment elevation myocardial infarction.

Table 10. Baseline characteristics of the ACS population and the propensity-matched subpopulation of NSTEMI and STEMI patients according to recreational drug detection.

	**Overall ACS population (N=712)**	**Propensity-matched NSTEMI population (N=197)**			**Propensity-matched STEMI population (N=197)**		
		**No recreational drug detected (N=169)**	**Recreational drug detected (N=28)**	**p-value**	**No recreational drug detected (N=174)**	**Recreational drug detected (N=23)**	**p-value**
Age, years	64±13	63 ± 12	56 ± 11	**0.001**	65 ± 12	48 ± 12	**<0.001**
Men, n (%)	531 (74.6)	133 (78.7)	25 (89.3)	0.193	127 (73.0)	20 (87.0)	0.148
Body mass index, kg/m²	27.3 ± 5.2	27.2 ± 4.9	27.0 ± 6.4	0.284	27.6 ± 5.2	26.1 ± 5.1	0.072
**CV risk factors**, n (%)							
Hypertension	394 (55.3)	84 (49.7)	8 (28.6)	**0.038**	98 (56.3)	5 (21.7)	**0.002**
Diabetes	162 (22.8)	37 (21.9)	2 (7.1)	0.070	38 (21.8)	2 (8.7)	0.175
Dyslipidemia	286 (40.2)	54 (32.0)	2 (7.1)	**0.007**	58 (33.3)	8 (34.8)	0.890
Family history of CAD	151 (21.2)	35 (20.7)	5 (17.9)	0.728	38 (21.8)	4 (17.4)	0.789
Smoking	231 (32.4)	55 (32.5)	21 (75.0)	**<0.001**	52 (29.9)	16 (69.6)	**<0.001**
**Medical history of CV disease**, n (%)							

History of ACS	110 (15.4)	19 (11.2)	3 (10.7)	>0.999	18 (10.3)	3 (13.0)	0.718
Previous PCI	429 (60.3)	125 (74.0)	20 (71.4)	0.778	117 (67.2)	16 (69.6)	0.823
History of coronary disease[a]	491 (69.0)	132 (78.1)	20 (71.4)	0.436	126 (72.4)	18 (78.3)	0.552
History of CKD[b]	51 (7.2)	16 (9.5)	2 (7.1)	>0.999	9 (5.2)	0 (0.0)	0.602
History of CV disease[c]	516 (72.5)	135 (79.9)	21 (75.0)	0.556	131 (75.3)	18 (78.3)	0.755
Alcohol consumption	403 (57.6)	105 (62.9)	17 (60.7)	0.827	102 (61.1)	15 (65.2)	0.702
**Clinical data on admission**							
Systolic blood pressure, mm Hg	136 ± 26	141 ± 26	136 ± 23	0.309	129 ± 24	120 ± 15	0.071
Heart rate, beats/min	79 ± 18	77 ± 17	88 ± 20	**0.002**	79 ± 18	86 ± 20	0.156
Oxygen saturation, %	97.3 ± 4.4	97.4 ± 2.9	97.4 ± 2.4	0.946	97.4 ± 2.7	97.7 ± 1.9	0.734
ICCU hospitalization duration, days	6.9 ± 15.8	5.5 ± 5.2	5.1 ± 3.8	0.844	7.9 ± 22.1	5.4 ± 2.7	0.846
Killip class				0.478			0.699
1	649 (91.3)	152 (89.9)	27 (96.4)		158 (91.3)	22 (95.7)	
≥ 2	62 (8.7)	17 (10.1)	1 (3.6)		15 (8.7)	1 (4.3)	
CHF signs	123 (17.3)	29 (17.2)	3 (10.7)	0.581	29 (16.7)	3 (13.0)	>0.999
**Laboratory results**							
Hemoglobin, g/dL	13.9 ± 1.8	14.2 ± 1.6	14.5 ± 1.8	0.218	13.9 ± 1.8	14.4 ± 1.8	0.356
Creatininemia, μmol/L	91 ± 64	99 ± 83	78 ± 29	0.214	90 ± 69	78 ± 18	0.930
High-sensitivity cardiac troponin	638 ± 1523	262 ± 548	306 ± 866	0.447	673 ± 2183	368 ± 473	0.717

peak[d]							
BNP, pg/mL	289 ± 613	338 ± 733	178 ± 358	0.388	294 ± 634	46 ± 30	0.189
**Echocardiography data**							
LV ejection fraction, %	53 ± 11	55 ± 11	55 ± 8	0.442	51 ± 11	49 ± 12	0.433
TAPSE, mm	21.5 ± 4.1	21.4 ± 3.8	23.2 ± 5.2	0.125	21.6 ± 4.1	19.4 ± 3.1	**0.029**
sPAP, mmHg	32 ± 12	30 ± 13	35 ± 9	**0.010**	29 ± 11	40 ± 14	**0.002**
TAPSE/sPAP ratio	0.8 ± 0.4	0.8 ± 0.4	0.7 ± 0.3	0.547	0.8 ± 0.4	0.6 ± 0.2	**<0.001**
VTI, cm	19.4 ± 4.8	20.2 ± 4.9	18.5 ± 5.2	0.060	19.2 ± 4.4	17.4 ± 4.2	0.066
E/e' ratio	8.3 ± 3.0	7.86 ± 2.65	7.78 ± 3.44	0.635	8.5 ± 3.1	7.0 ± 2.2	**0.046**
E/A ratio	1.0 ± 0.5	1.0 ± 0.4	1.1 ± 0.5	0.121	1.0 ± 0.4	1.1 ± 0.5	0.307

Values are n (%), mean ± SD, or median (interquartile range).

[a]*History of coronary disease defined by the presence of ³1 of the following: ACS, angioplasty, or coronary bypass.*

[b]*CKD defined by history of chronic kidney disease with glomerular filtration rate <60 mL/min/m^2.*

[c]*CV disease defined by the presence of known MI, previous PCI, previous CABG, peripheral atheroma with revascularization, stroke, history of heart failure, history of atrial fibrillation, history of surgery for valvular heart disease, pacemaker or ICD, and cardiomyopathies.*

[d]*Represents the n-fold troponin levels.*

<u>Abbreviations</u>: *ACS: acute coronary syndrome; BNP: B-type natriuretic peptide; CABG: coronary artery bypass graft; CAD: coronary artery disease; CHF: congestive heart failure; CKD: chronic kidney disease; CV: cardiovascular; ICCU: intensive cardiac care unit; ICD: Implantable Cardioverter-Defibrillator; LV: left ventricle; MI: myocardial infarction; NSTEMI: non-ST-segment elevation myocardial*

infarction; PCI: percutaneous coronary intervention; SD: standard deviation; sPAP: systolic pulmonary artery pressure; STEMI: ST-segment elevation myocardial infarction; TAPSE: tricuspid annular plane systolic excursion; VTI: velocity time integral.

Figures

Figure 1. Presentation of the urine drug test

The following psychoactive drugs were detected using a multidrug test (NarcoCheck®, Kappa City Biotech SAS, Montluçon, France): i) cannabinoids (tetrahydrocannabinol [THC]), including cannabis and hashish; ii) cocaine and metabolites, including cocaine and crack; iii) amphetamines; iv) MDMA; and v) heroin and other opioids.

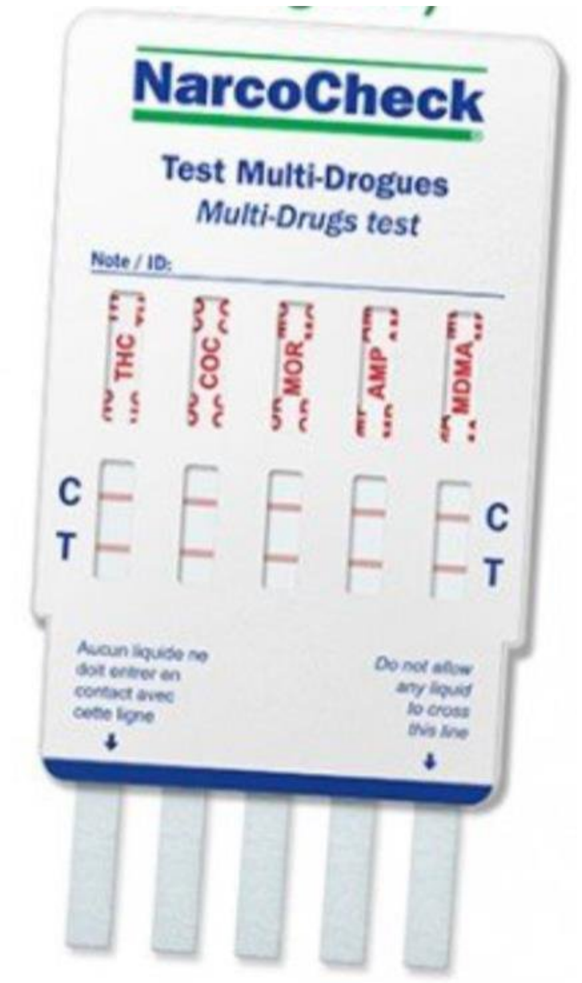

References

1. Collet JP, Thiele H, Barbato E, et al. 2020 ESC Guidelines for the management of acute coronary syndromes in patients presenting without persistent ST-segment elevation. *Eur Heart J*. 2021;42(14):1289-1367. doi:10.1093/eurheartj/ehaa575
2. Ibanez B, James S, Agewall S, et al. 2017 ESC Guidelines for the management of acute myocardial infarction in patients presenting with ST-segment elevation: The Task Force for the management of acute myocardial infarction in patients presenting with ST-segment elevation of the European Society of Cardiology (ESC). *Eur Heart J*. 2018;39(2):119-177. doi:10.1093/eurheartj/ehx393
3. McDonagh TA, Metra M, Adamo M, et al. 2021 ESC Guidelines for the diagnosis and treatment of acute and chronic heart failure. *Eur Heart J*. 2021;ehab368. doi:10.1093/eurheartj/ehab368
4. Friedrich MG, Sechtem U, Schulz-Menger J, et al. Cardiovascular magnetic resonance in myocarditis: A JACC White Paper. *J Am Coll Cardiol*. 2009;53(17):1475-1487. doi:10.1016/j.jacc.2009.02.007
5. Ghadri JR, Wittstein IS, Prasad A, et al. International Expert Consensus Document on Takotsubo Syndrome (Part II): Diagnostic workup, outcome, and management. *Eur Heart J*. 2018;39(22):2047-2062. doi:10.1093/eurheartj/ehy077
6. Konstantinides SV, Meyer G, Becattini C, et al. 2019 ESC Guidelines for the diagnosis and management of acute pulmonary embolism developed in collaboration with the European Respiratory Society (ERS). *Eur Heart J*. 2020;41(4):543-603. doi:10.1093/eurheartj/ehz405
